# A bibliometric analysis of research on the learning environment in medical schools: trends, gaps, and global perspectives

**DOI:** 10.3389/fmed.2026.1760995

**Published:** 2026-02-03

**Authors:** Afaf Sulaiman Alblooshi, Falah Mohammed Almarzooqi, Anmol Punn, Taleb M. Almansoori, Raghad Salem Alharthi, Shaikha Ahmed Alzaabi, Gamila Ahmed, Saif Al-Shamsi, Faten Abdullah AlRadini

**Affiliations:** 1 Department of Medical Education, College of Medicine and Health Sciences, United Arab Emirates (UAE) University, Al Ain, Abu Dhabi, United Arab Emirates; 2 Department of Family Medicine, College of Medicine and Health Sciences, United Arab Emirates (UAE) University, Al Ain, Abu Dhabi, United Arab Emirates; 3 Department of Radiology, College of Medicine and Health Sciences, United Arab Emirates (UAE) University, Al Ain, Abu Dhabi, United Arab Emirates; 4 College of Medicine and Health Sciences, United Arab Emirates (UAE) University, Al Ain, Abu Dhabi, United Arab Emirates; 5 Public Services and Outreach Unit, National Medical Library, College of Medicine and Health Sciences, United Arab Emirates (UAE) University, Al Ain, Abu Dhabi, United Arab Emirates; 6 Department of Internal Medicine, College of Medicine and Health Sciences, United Arab Emirates (UAE) University, Al Ain, Abu Dhabi, United Arab Emirates; 7 Family and Community Medicine Department, College of Medicine, Princess Nourah bint Abdulrahman University, Riyadh, Saudi Arabia

**Keywords:** curriculum, educational climate, learning environments, medical education, medical school learning environment

## Abstract

**Introduction:**

The learning environment (LE) is widely recognized as a key focus of medical education scholarship, influencing student motivation, academic performance, professional identity formation, and wellbeing. Despite its recognized importance, a global, comprehensive understanding of research trends, key contributors, and thematic evolution within medical school learning environment (MSLE) research remains limited.

**Methods:**

We conducted a bibliometric analysis of 3,458 publications on MSLE published between 2000 to 2025, sourced from Web of Science, Scopus, and PubMed. The Bibliometrix R package was used for network visualization and bibliometric analyses, examining publication trends, leading countries, institutions, journals, authors, and keywords. Keyword co-occurrence and thematic mapping was used to explore major research themes and their evolution over time.

**Results:**

MSLE research has expanded steadily over the past two decades, with contributions from diverse global regions. Leading institutions and journals continue to anchor the field, while emerging topics such as program evaluation, digital pedagogy, and student wellbeing reflect shifting scholarly priorities in medical education. Prominent keyword clusters relate to curriculum, medical education, medical student, learning environment, and perception, demonstrating a balance between foundational and specialized research. Thematic mapping identified central, well-developed domains such as professionalism and program evaluation, alongside emerging and niche topics like online learning and simulation, highlighting both ongoing maturation and diversification of the field.

**Conclusion:**

This bibliometric analysis provides a structured overview of global MSLE research, illustrating publication trends, influential contributors, and evolving thematic priorities. The findings indicate that the learning environment has become a central focus of medical education research and highlight the need for greater emphasis on longitudinal, multi-institutional studies, international collaboration, and inclusion of underrepresented regions to inform future research and policy development.

## Introduction

The learning environment (LE) is a key factor in shaping and training future physicians. The LE encompasses the physical, social, and psychological settings where students learn, acquire knowledge, and it is shaped by their interactions with peers, staff, faculty, curriculum, resources and the infrastructure around them (1). A positive LE has been linked to higher levels of motivation, academic achievement, empathy, and professional identity formation, while negative LE have been associated with burnout, disillusionment, and reduced academic performance (2–6). Thus, understanding the LE is essential for ongoing program evaluation and a critical step toward educational reform in medical schools (7–9).

In recognition of its influence, medical education accreditation bodies worldwide have emphasized the need for continuous assessment and enhancement of the LE (10–13). This focus has translated into calls for structured evaluation tools and regular feedback mechanisms to guide curriculum development, enhance faculty teaching practices, and improve student wellbeing and support services. The Liaison Committee on Medical Education (LCME) which is the accrediting authority for medical schools in the U.S. and Canada, requires institutions to ensure that their learning environments promote appropriate professional behavior, student wellbeing, and respectful treatment of all individuals. The LCME also mandates periodic evaluations to identify both positive and negative influences and to implement strategies that strengthen professionalism and student development across all clinical and academic settings (14). This highlights a system-wide expectation for medical schools to actively monitor and constantly improve their educational environments. To facilitate these evaluations, several validated instruments have been developed to assess students’ perceptions of the LE in medical schools. Among the most commonly used are the Dundee Ready Education Environment Measure (DREEM), the Johns Hopkins Learning Environment Scale (JHLES), and the Medical School Learning Environment Survey (MSLES) (15–17). These instruments measure multiple dimensions of the LE, including academic atmosphere, social support, perceptions of teachers, and emotional wellbeing. Their widespread adoption in studies from countries such as United States, United Kingdom, Australia, Canada and Netherlands underscores their utility in capturing student experiences across diverse cultural and institutional (4, 18–23).

Despite the growing body of research on this topic, there remains a lack of comprehensive analysis of how the domain of learning environment research has evolved globally over time. While individual studies provide insights into specific contexts, there is limited understanding of the broader trends, key contributors, and emerging themes that characterize LE field. This is where a bibliometric analysis becomes especially valuable. It provides a systematic and quantitative approach to examining large volumes of scholarly literature, allowing researchers to map publication growth, identify influential authors, institutions, and journals, and explore the evolution of research themes over time. Unlike narrative or systematic reviews, bibliometric methods are particularly effective for revealing structural patterns and research gaps across a field, making them well suited to identifying structural patterns and research gaps across a field (24). Importantly, this study examines the evolution and structure of the research literature on medical school learning environments, rather than directly assessing or comparing the current learning environments of individual medical schools.

Given the LEs central role in shaping academic achievement, psychological health, and long-term professional conduct, it is essential to map and understand the scholarly landscape of research examining this topic. Accordingly, this study applies bibliometric methods to analyze publication growth, geographical distribution of research output, leading authors and institutions, journal sources, collaboration networks, and thematic trends over time. By providing a comprehensive overview of the MSLE research landscape, these findings can help identify gaps and opportunities for future investigation and support educators, researchers, policymakers, and funding bodies in strengthening research and innovation in this domain (24).

## Methods

### Study design

This study employs a bibliometric analysis approach, using bibliometric indicators to quantitatively assess the research landscape of scholarship on the Learning Environment in Medical Schools.

### Data sources and search strategy

Data for this study were extracted from three major citation databases Web of Science (WoS) Core Collection, Scopus, and PubMed on the 17th of July 2025 by a medical librarian. The search strategy was developed using Medical Subject Headings (MeSH) and title/abstract fields to ensure comprehensive literature retrieval. The keywords related to Learning Environment included are:

- “Learning Environment”
- “Medical schools”

Search strategies were customized for each database, with an English language filter applied. A detailed description of the search terms and strategy is provided in Supplementary material 1. Additionally, reference lists of included studies were manually screened for further eligible articles. Grey literature was excluded to maintain the quality and rigor of the analysis. The search was restricted to articles published between 2000 and 2025 (mid-year), ensuring coverage of recent advancements in MSLE literature. Only peer-reviewed journal articles and conference proceedings were included, while book chapters, editorials, and non-peer-reviewed sources were excluded.

### Inclusion and exclusion criteria

The inclusion criteria for this study were publications explicitly focusing on the LE in medical education within medical schools, and articles indexed in Web of Science, Scopus, or PubMed with complete citation records available. Exclusion criteria included non-English publications, studies not related to the LE in medical education, and non–peer-reviewed sources such as editorials, commentaries, or conference abstracts without full text. Additionally, studies focusing on non-medical disciplines or on postgraduate medical education were excluded.

### Data synthesis and analysis

Quantitative analysis was conducted using the Bibliometrix package (version 5.1.0) in R software (version 4.4.1; R Foundation for Statistical Computing, Vienna, Austria). The following variables were extracted and analyzed through the package: publication year (to track research trends over time), citation count (to assess impact), authors and affiliations (to identify leading researchers and institutions), journals (to determine where high-impact research is published), and keywords and thematic trends (to map the research focus over time). To illustrate key research trends and knowledge gaps, the following analyses were conducted: co-authorship analysis (mapping collaboration networks among institutions, researchers and countries), keyword co-occurrence analysis (identifying emerging themes, evolving research topics, and research gaps), and citation analysis (highlighting the most influential publications and authors in MSLE research).

Keyword based thematic analyses were conducted using author-supplied keywords extracted from the bibliographic records in Web of Science Scopus and PubMed. No selective exclusion of author-provided keywords was applied in order to preserve the original structure of the metadata and ensure reproducibility of the bibliometric outputs. The thematic structure of the literature was explored using keyword co-occurrence analysis visualized through a word cloud and a thematic map generated using the Bibliometrix package. Highly frequent terms reflect common author keyword and indexing practices and were therefore interpreted in context with emphasis placed on conceptually meaningful educational themes relevant to MSLE research.

### Ethical considerations

This study involved secondary data analysis of publicly available literature, and no ethical approval was required. All data were retrieved from reputable databases, ensuring compliance with publication standards.

## Results

**Figure 1:**
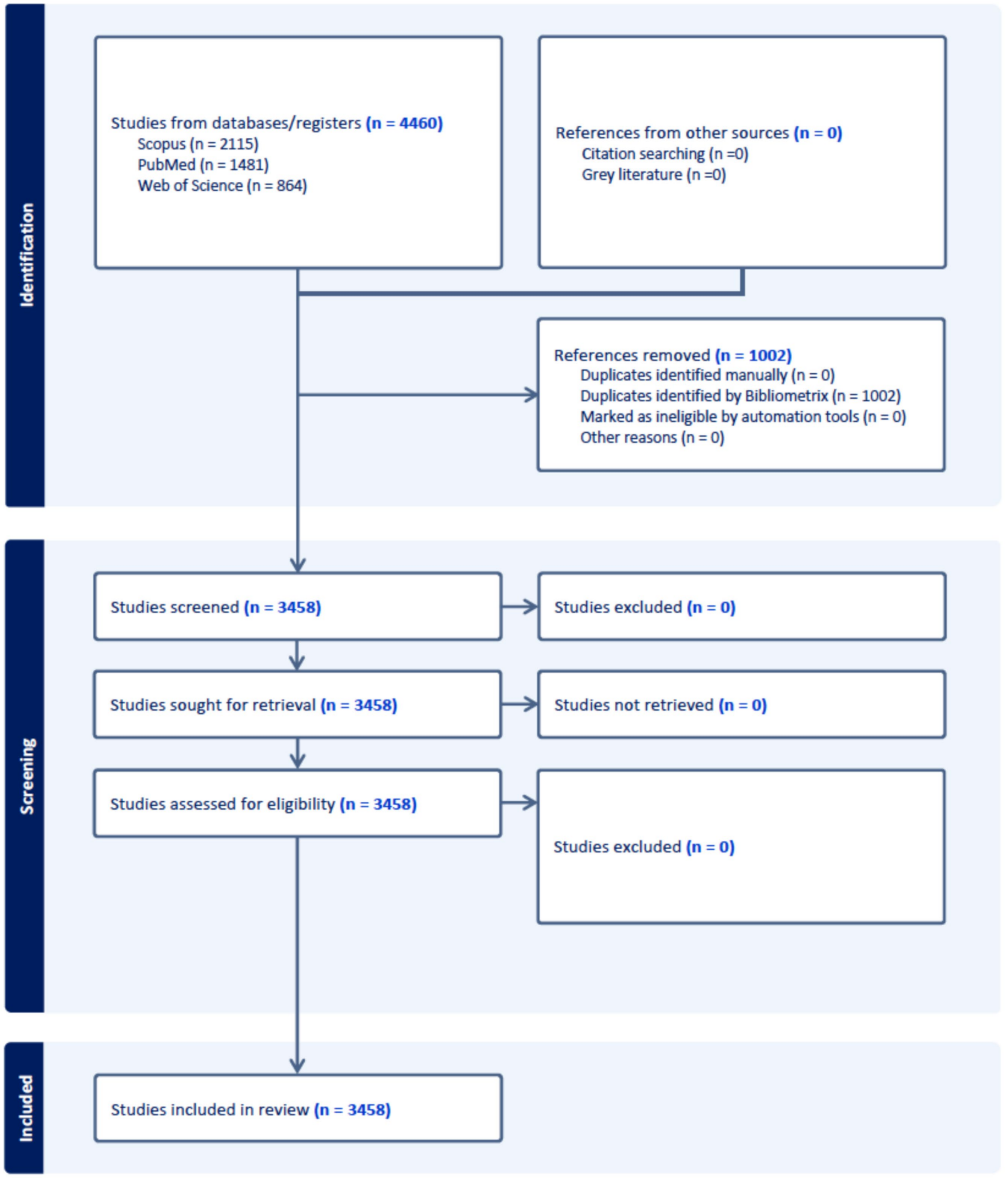
PRISMA flow diagram illustrating the identification, de-duplication, and inclusion of records for the bibliometric analysis of research on the medical school learning environment.

The database search retrieved 4,460 records. Following the removal of 1,002 duplicates using Bibliometrix tool, 3,458 unique records were retained for screening (Figure 1).

### Annual trends in scientific production

**Figure 2:**
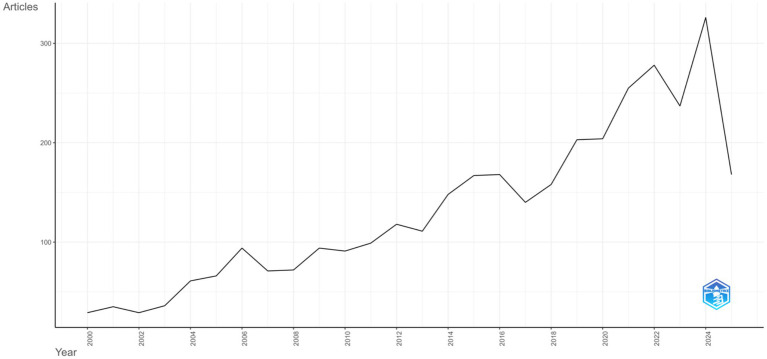
Annual scientific production from 2000 to 2025.

The annual scientific production in the field of LE research demonstrated a clear upward trend over the study period (Figure 2). Between 2000 and 2005, the number of publications remained modest, ranging from 29 to 66 articles per year. From 2006 onward, output increased to 94 articles and showed gradual fluctuations with an overall upward trajectory in subsequent years. From 2010, steady growth was observed, with publications reaching 148 in 2014 and peaking at 204 in 2020. The upward momentum continued in recent years, with 278 articles in 2022 and the highest output recorded in 2024 with 326 publications. The apparent decline in 2025 (168 publications) is likely explained by partial data availability, as data extraction was conducted in July 2025.

**Figure 3:**
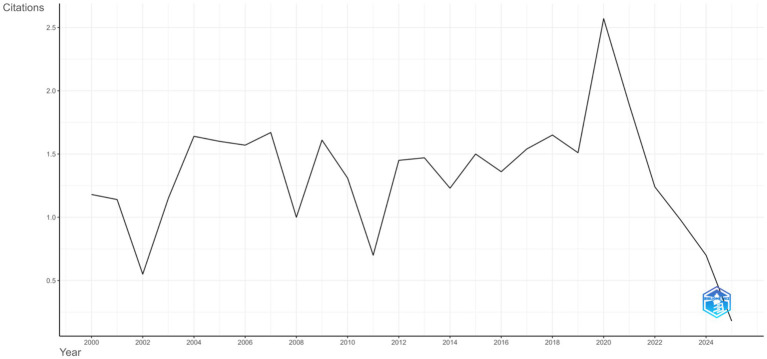
Average citations per year.

The average number of citations per article per year showed relative stability between 2000 and 2019, fluctuating between 0.7 and 1.7 citations annually. A pronounced peak was observed in 2020, when the average exceeded 2.5 citations per article, followed by a sharp decline, with values dropping below 0.5 citations in 2024 and 2025. The 2025 data reflect the mid-year extraction point (July 2025). Figure 3 illustrates these citation trends over time.

### Geographical distribution of research output

**Figure 4:**
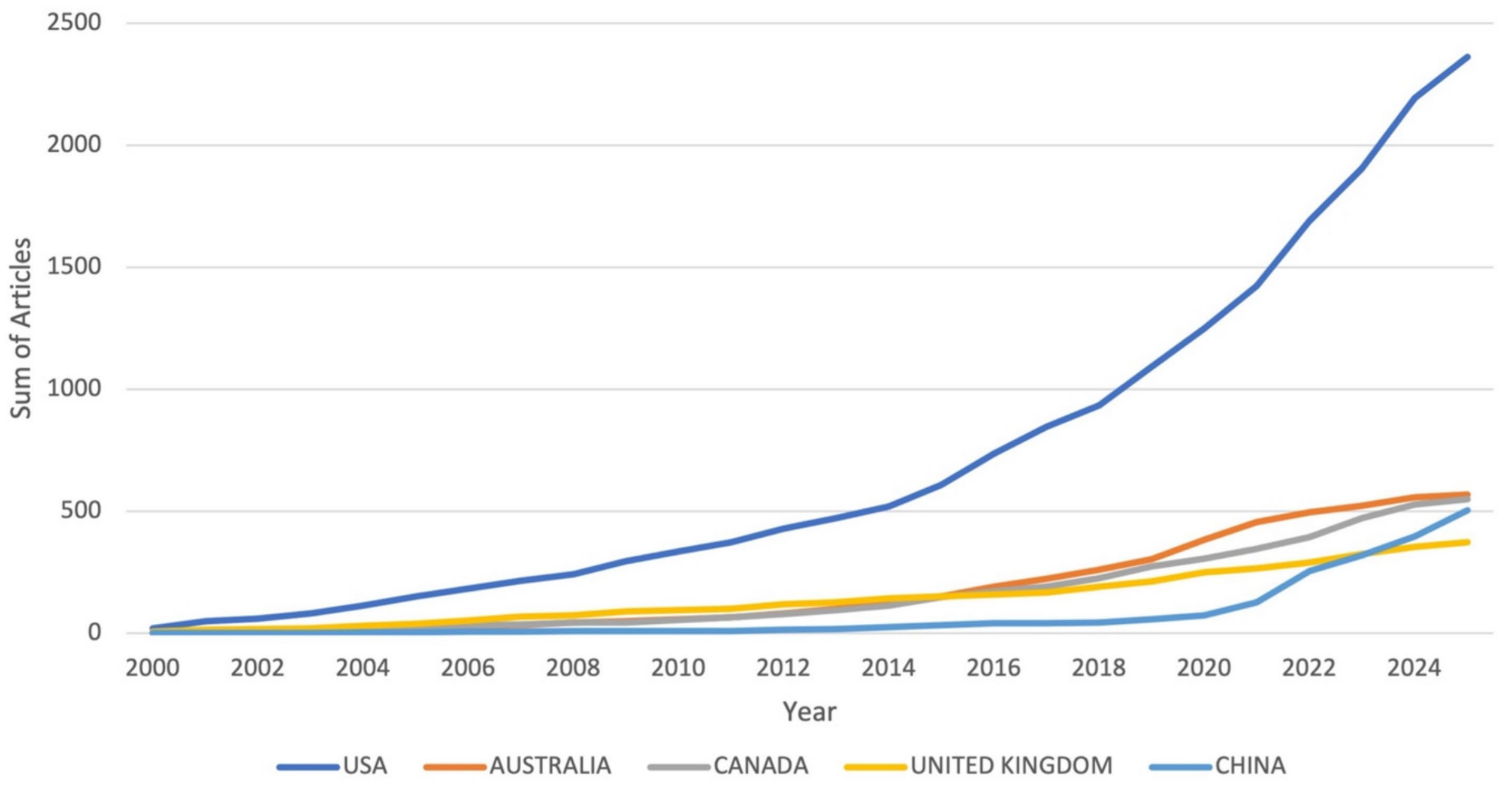
Country production over time.

**Figure 5:**
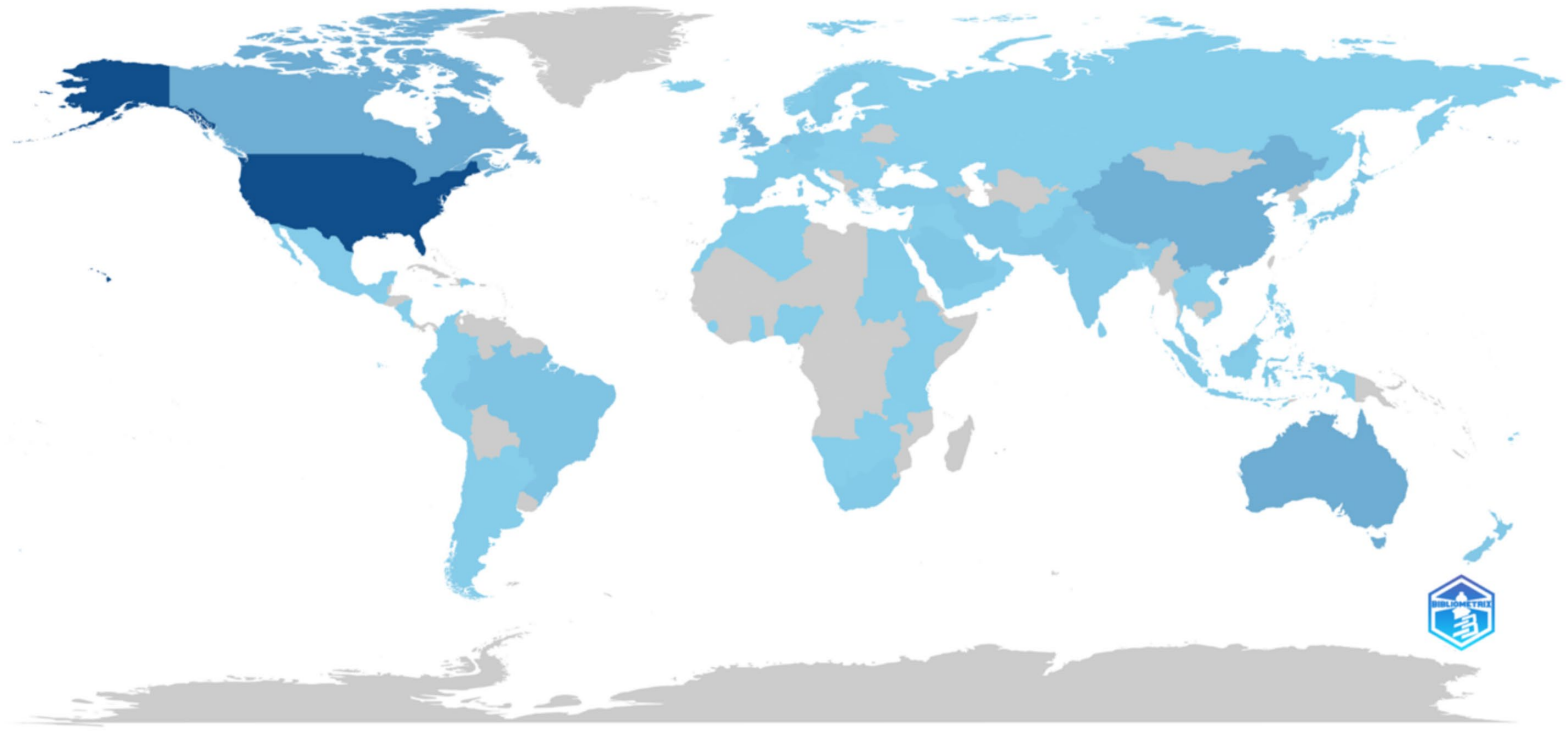
Country scientific production.

Analysis of country-level scientific production shows that the United States contributed the highest number of publications (*n* = 2,364), followed by Australia (*n* = 569), Canada (*n* = 550), China (*n* = 503), and the United Kingdom (*n* = 372) (Supplementary material 4). Other notable contributors included the Netherlands (*n* = 281), Singapore (*n* = 189), Brazil (*n* = 172), Pakistan (*n* = 167), Iran (*n* = 141), Japan (*n* = 128), Saudi Arabia (*n* = 128), Germany (*n* = 127), Ireland (*n* = 116), and India (*n* = 113). The line graph depicting country production over time in Figure 4 highlights that the United States consistently maintained the highest output across all years, with steady growth particularly after 2010. Australia and Canada also demonstrated gradual increases, while China exhibited a marked rise in later years, positioning itself among the leading contributors. The United Kingdom showed a progressive upward trend, although at comparatively lower levels than the other top-producing countries. The global map of scientific production further illustrates these patterns, with darker shading observed in high-output countries such as the United States, Australia, Canada, China, and the United Kingdom, while lighter shading represents countries with lower levels of publication activity (Figure 5).

A focused analysis of GCC (Gulf Cooperation Council) countries reveals distinct patterns of scholarly output in the field of learning environment research (Supplementary material 5). Saudi Arabia leads regional production with 128 publications, followed by the United Arab Emirates (52), Qatar (21), Oman (11), Kuwait (8), and Bahrain (2). This data highlights Saudi Arabia as the predominant GCC contributor to the literature, with a substantial margin compared to its regional counterparts.

### Analysis of journals and sources

The journals contributing the highest number of articles on MSLE research are led by BMC Medical Education with 293 publications. Medical Teacher follows with 173 articles, while Academic Medicine and Medical Education contributed 115 and 114 articles, respectively. Nursing-focused journals were also represented, alongside other notable sources such as the Journal of Surgical Education, Teaching and Learning in Medicine, and the Journal of Graduate Medical Education, reflecting contributions from a range of health professions and educational contexts (Table 1; Supplementary material 10).

**Table 1:** Most relevant sources.

S.No.	Sources	Articles
1	BMC MEDICAL EDUCATION	293
2	MEDICAL TEACHER	173
3	ACADEMIC MEDICINE	115
4	MEDICAL EDUCATION	114
5	NURSE EDUCATION TODAY	112
6	ACADEMIC MEDICINE: JOURNAL OF THE ASSOCIATION OF AMERICAN MEDICAL COLLEGES	74
7	NURSE EDUCATION IN PRACTICE	56
8	JOURNAL OF SURGICAL EDUCATION	54
9	TEACHING AND LEARNING IN MEDICINE	45
10	JOURNAL OF GRADUATE MEDICAL EDUCATION	37
11	BMJ OPEN	36
12	MEDICAL EDUCATION ONLINE	36
13	JOURNAL OF DENTAL EDUCATION	34
14	ADVANCES IN MEDICAL EDUCATION AND PRACTICE	31
15	JOURNAL OF GENERAL INTERNAL MEDICINE	30

Among the top journals ranked by local h-index (within corpus), Medical Teacher stands out with the highest score of 42, reflecting consistent and high-quality publication output. Academic Medicine (local h-index: 35) and Medical Education (local h-index: 33) also demonstrate long-established influence in the field. BMC Medical Education, though relatively newer (established in 2005), has achieved a local h-index of 31, underscoring its growing prominence and open-access reach. Nurse Education Today follows with a local h-index of 26, highlighting the increasing role of nursing education in learning environment research. Other journals, including Advances in Health Sciences Education, Medical Education Online, and the Journal of General Internal Medicine, exhibit impact with local h-indices ranging from 12 to 15 (Supplementary material 11).

**Figure 6:**
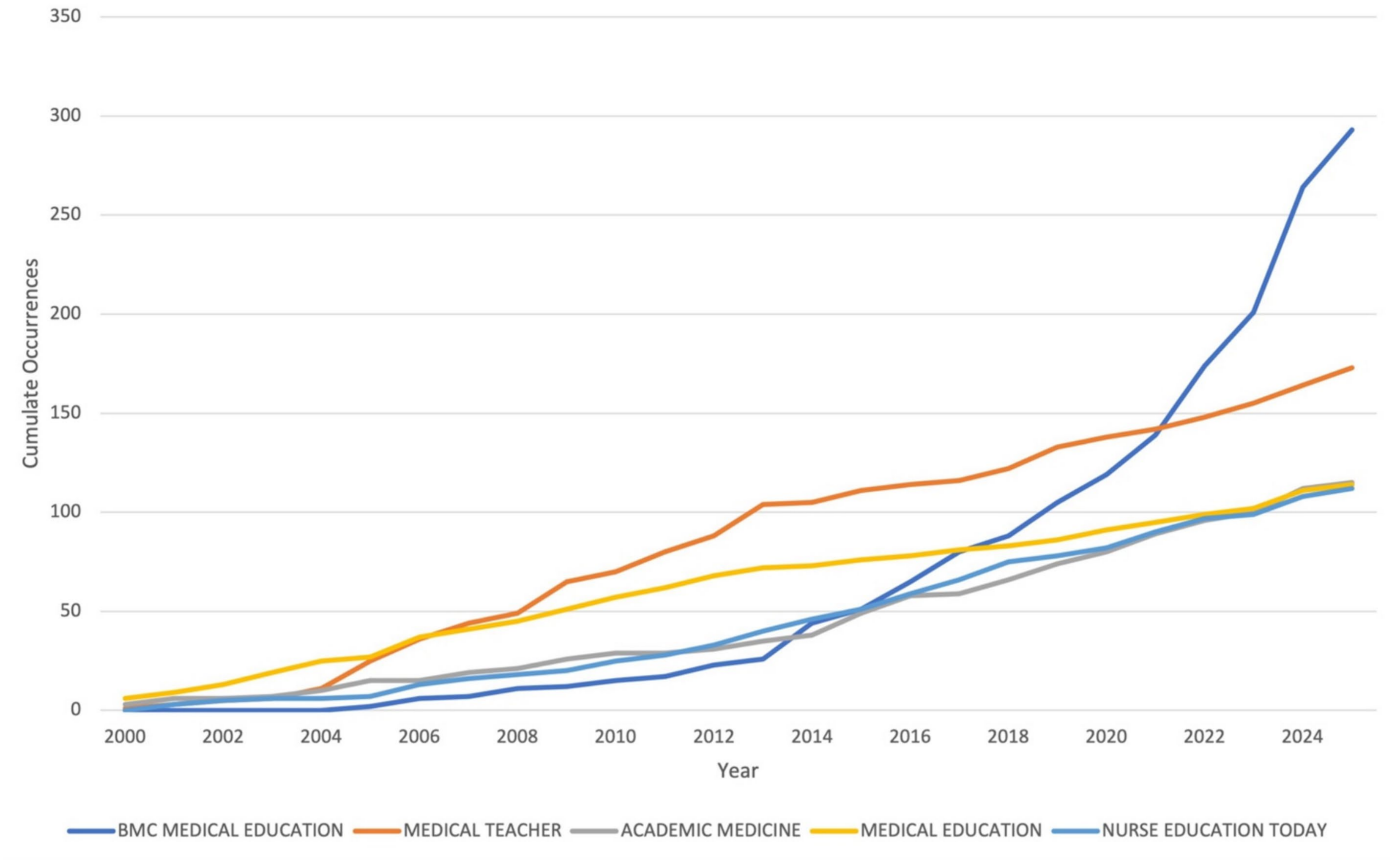
Sources’ production over time.

Reviewing the publication trends over the years for five major journals, all journals show a steady increase in publication volume over time, reflecting the growing research interest in the topic (Figure 6). BMC Medical Education shows the most dramatic rise from no articles in 2000 to 293 in 2025, signaling a sharp upward trend particularly from 2015 onwards. Medical Teacher maintains consistent growth, reaching 173 articles by 2025. Academic Medicine and Medical Education both show a more modest but steady increase, reaching 115 and 114 articles, respectively. Nurse Education Today also displays consistent growth, peaking at 112 articles in 2025. This pattern reflects an overall surge in research output in this field, particularly over the past decade, possibly linked to growing global attention on improving educational quality and student wellbeing in health professions education.

### Authors analysis

**Figure 7:**
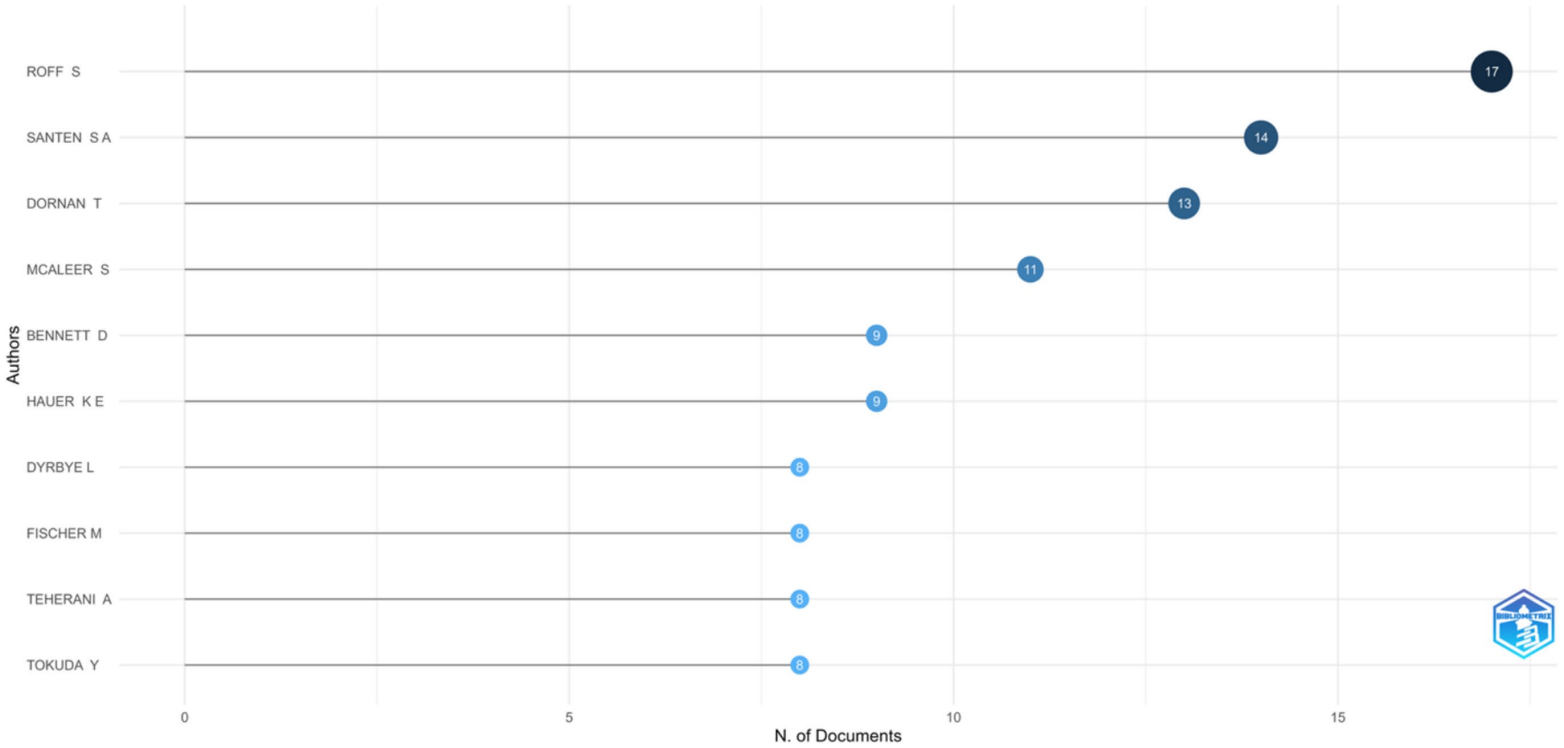
Most relevant authors.

The leading authors based on the number of published articles in learning environment research within medical education are summarized in Figure 7. Roff S leads with 17 articles, followed by Santen S A (14 articles) and Dornan T (13 articles). Several authors including Mcaleer S, Bennett D, Hauer K E, Dyrbye L, Fischer M, Teherani A, Tokuda Y, Van D V C, and Watling C have contributed between 8 and 11 articles each (Supplementary material 15). This distribution highlights a group of consistently productive scholars contributing to the advancement of research on the learning environment in medical education.

The most influential authors within the analyzed dataset, based on the h-index, are presented in Supplementary material 16. This metric reflects both productivity and citation impact. Roff S leads with an h-index of 13, while McAleer S and Dornan T follow closely with h-indices of 10 and 9, respectively, and are all noted contributors to foundational literature on the MSLE. Several authors including Bennett D, Santen S A, Hauer K E, and Watling C, have h-indices ranging between 7–8, indicating consistent publication of well-cited work. Emerging contributors such as Appelbaum N P and Chang Y also show promising local impact with h-indices of 6, demonstrating rapidly growing influence (Supplementary material 17). These findings highlight a combination of long-standing pioneers and newer voices shaping scholarly discourse in the domain of medical education learning environments.

The author collaboration map in Supplementary material 18 highlights distinct clusters of researchers contributing to field. Notable clusters are centered around prolific authors such as Santen S A, Dornan T, and Roff S, each of whom is connected with smaller networks of collaborators. While certain groups (e.g., Dornan’s cluster) show stronger internal connectivity, overall inter-cluster collaboration remains limited, suggesting that research activity is still organized around regionally or thematically concentrated groups of scholars.

### Institutional contributions

**Figure 8:**
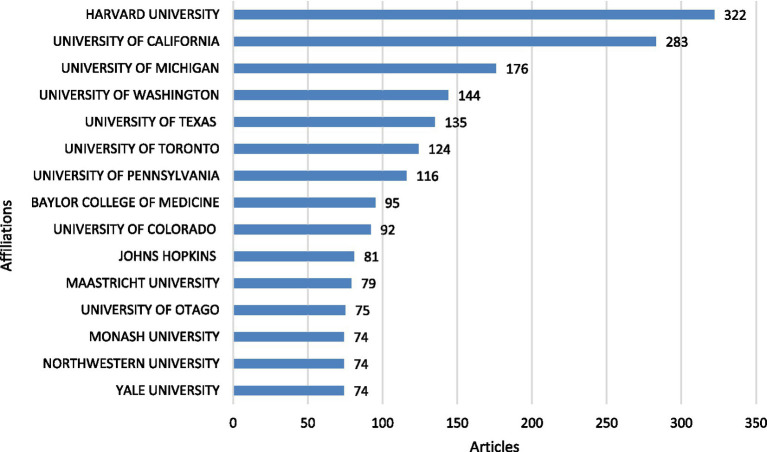
Most relevant affiliations.

The analysis of leading institutional affiliations in MSLE research shows that Harvard University is the most prolific, contributing 322 articles. It is followed by the University of California with 283 articles and the University of Michigan with 176 articles. Other highly productive institutions include the University of Washington, University of Texas, and University of Toronto, while the University of Pennsylvania, Baylor College of Medicine, University of Colorado, and Johns Hopkins University also demonstrate substantial publication output (Figure 8; Supplementary material 20). This distribution highlights the varying levels of institutional productivity in MSLE research, with a small number of affiliations accounting for a significant proportion of scholarly output in the field.

### Country-level collaboration and impact

**Figure 9:**
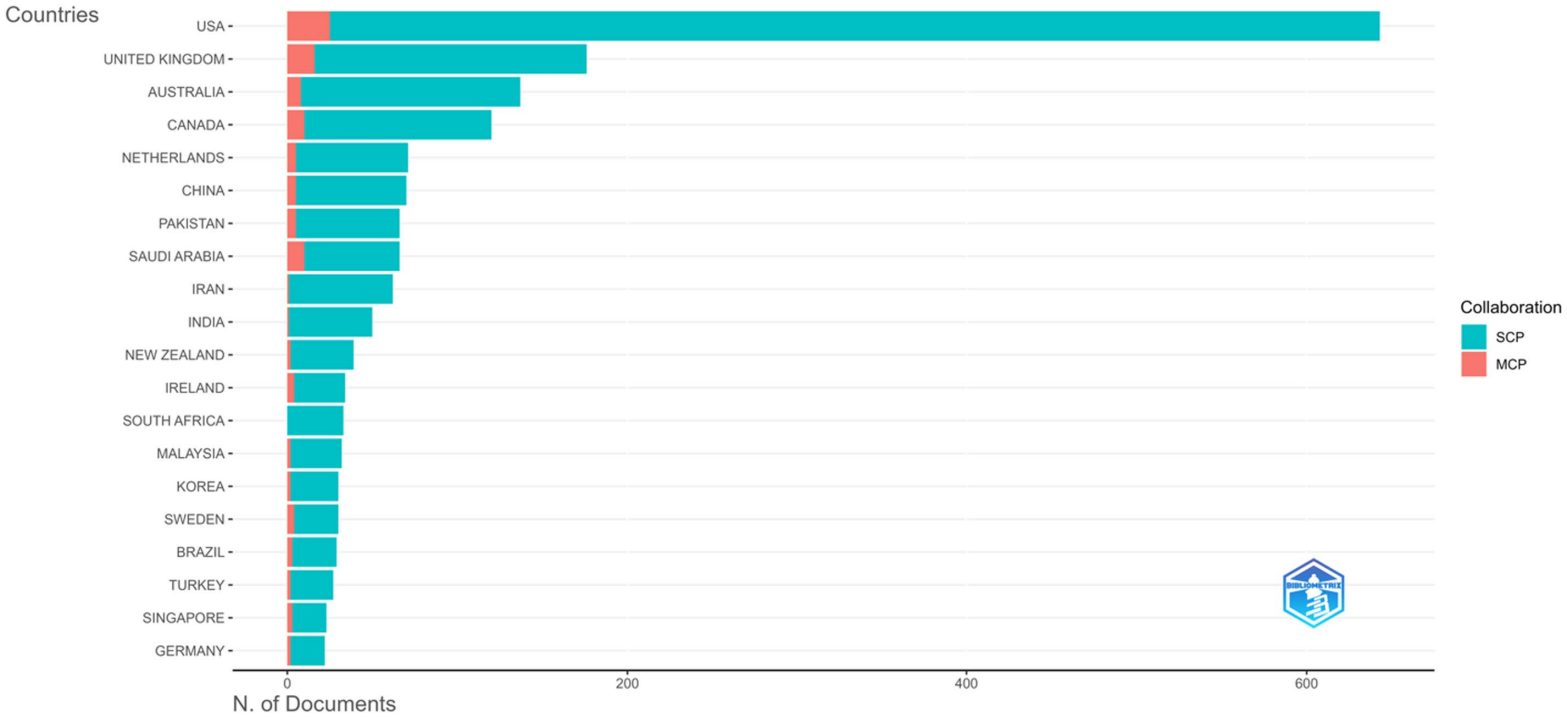
Corresponding author countries.

The analysis of corresponding authorship reveals the most productive countries in research on the learning environment in medical education within medical schools (Figure 9). The United States leads significantly with 643 articles (18.6% of total), of which 618 are single-country publications (SCP) and 25 are multi-country publications (MCP), indicating a lower proportion of international collaboration (3.9%). The United Kingdom ranks second with 176 articles (5.1%), and a relatively higher MCP rate (9.1%), suggesting more active international collaboration. Australia (137 articles), Canada (120), and the Netherlands (71) follow in the list, with varying degrees of collaboration, Canada showing 8.3% MCP and the Netherlands 7%. Notably, Saudi Arabia has 66 corresponding-author articles with a 15.2% MCP rate, indicating strong international engagement relative to its output. Other prominent contributors include China, Pakistan, Iran, India, and Ireland, all showing differing levels of collaboration. South Africa, despite publishing 33 articles, had no international co-authorships, reflecting a solely domestic research focus (Supplementary material 9).

Analysis of the top 10 countries by total citations and average citations per article highlights the global distribution and impact of research in this field (Supplementary materials 7, 8). The United States leads with 12,060 citations, followed by the United Kingdom (6,300) and Canada (3,708). Notably, Israel achieved the highest average citations per article (42.2), despite having fewer publications. Other countries with high average citations include the UK (35.8), Canada (30.9), and New Zealand (28.3), reflecting the significant influence of their research outputs. Within the GCC region, Saudi Arabia recorded 986 total citations with an average of 14.9 per article, demonstrating both productivity and increasing visibility in the field.

**Figure 10:**
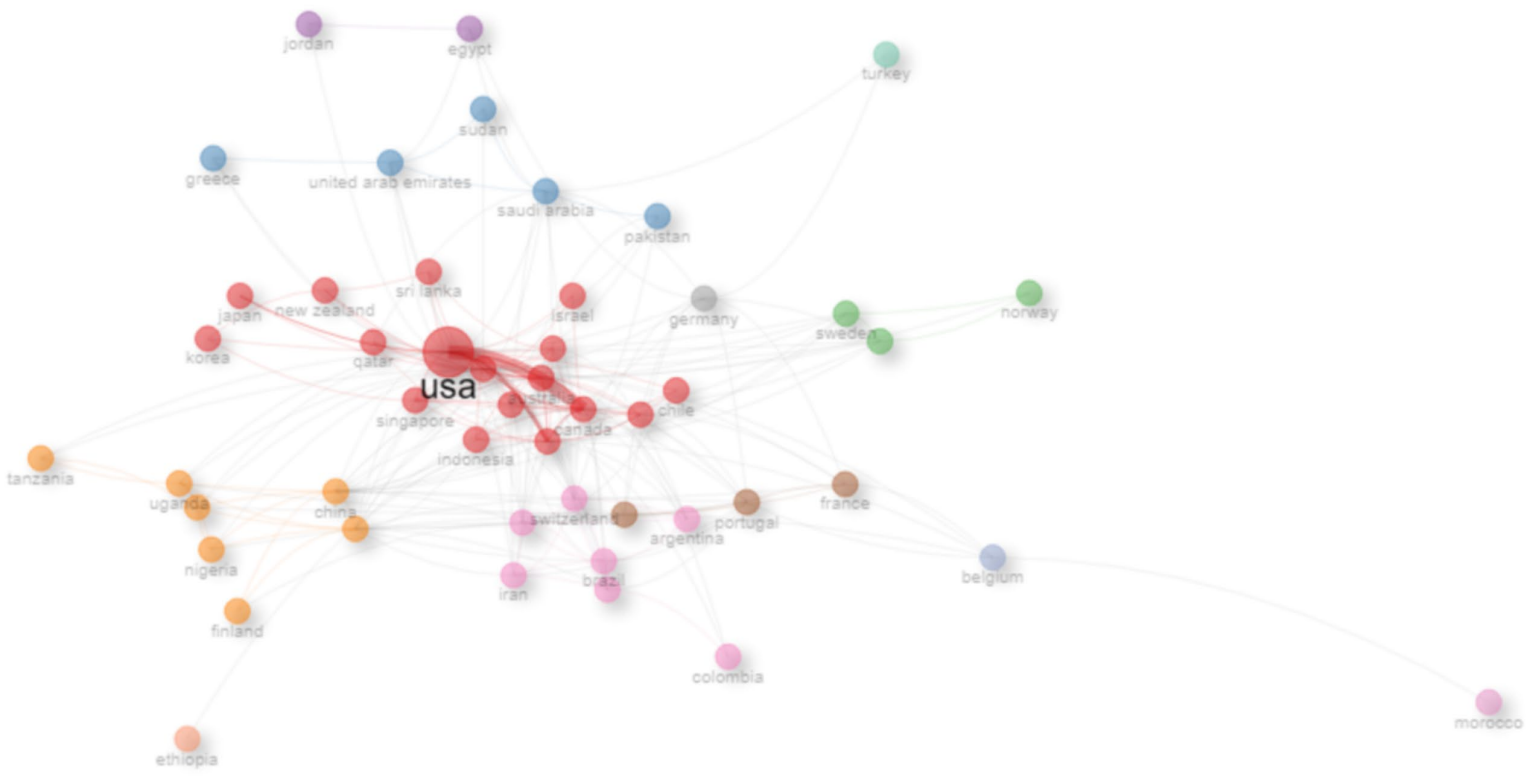
Country collaboration network.

The country-level collaboration network (Figure 10) illustrates strong partnerships, with the USA positioned at the center of global collaborations, showing extensive links with countries across Europe, Asia, and the Middle East. European countries such as the United Kingdom, Germany, and the Netherlands also exhibit strong connectivity, while emerging contributors from the Middle East (e.g., Saudi Arabia, UAE, Qatar) and Asia (e.g., China, Japan, Singapore) are increasingly integrated into the network. However, some regions (e.g., parts of Africa and South America) remain underrepresented, with sparse links observed.

### Highly cited publications

The most globally cited documents in the field of MSLE research, as identified by total citation counts, annual citation rates, and normalized citation metrics are presented in Table 2. The publication with the highest citation count is by Kamel B M N (2006) in BMC Medical Education, which has amassed 812 citations and maintains an average of 40.6 citations per year. This reflects sustained scholarly recognition over time. Swing S R (2007) in Medical Teacher follows closely, with 707 citations and 37.2 citations per year, highlighting its foundational role in discourse surrounding clinical education outcomes. Newer publications focusing on evolving educational methods, increased interest in digital and virtual learning solutions during and after the COVID-19 pandemic period, simulation-based education, clinical learning outcomes, and the influence of educational portfolios exhibit notable citation dynamics as well (Supplementary material 14). Most global cited documents, provides detailed citation metrics, including normalized citation counts, for each document.

**Table 2:** Top 10 most frequently cited documents.

No.	Author and journal	Year	Title of paper	Total citations	TC per year	Normalized TC
1	KAMEL B M N, BMC MED EDUC	2006	Wikis, blogs and podcasts: a new generation of Web-based tools for virtual collaborative clinical practice and education	812	40.6	25.8388626
2	SWING S R, MED TEACH	2007	The ACGME outcome project: retrospective and prospective	707	37.2105263	22.2603104
3	DOST S, BMJ OPEN	2020	Perceptions of medical students toward online teaching during the COVID-19 pandemic: a national cross-sectional survey of 2,721 UK medical students	481	80.1666667	31.2
4	MOULTON C, ANN SURG	2006	Teaching surgical skills: what kind of practice makes perfect?: a randomized, controlled trial	478	23.9	15.2105619
5	KYE B, J EDUC EVAL HEALTH P	2021	Educational applications of metaverse: possibilities and limitations	421	84.2	44.5826412
6	SCALESE R J, J GEN INTERN MED	2008	Simulation technology for skills training and competency assessment in medical education	335	18.6111111	18.5538462
7	BUCKLEY S, MED TEACH	2009	The educational effects of portfolios on undergraduate student learning: a Best Evidence Medical Education (BEME) systematic review. BEME Guide No. 11	313	18.4117647	11.4304584
8	HARDEN R, MED TEACH	2001	AMEE Guide No. 21: Curriculum mapping: a tool for transparent and authentic teaching and learning	309	12.36	10.8258258
9	CLELAND J, MED TEACH	2009	The use of simulated patients in medical education: AMEE Guide No 42	294	17.2941176	10.7365967
10	PAPP I, NURSE EDUC TODAY	2003	Clinical environment as a learning environment: student nurses’ perceptions concerning clinical learning experiences	288	12.5217391	10.9136842

DOI, Digital Object Identifier; TC, total citations.

### Thematic evolution of research

**Figure 11:**
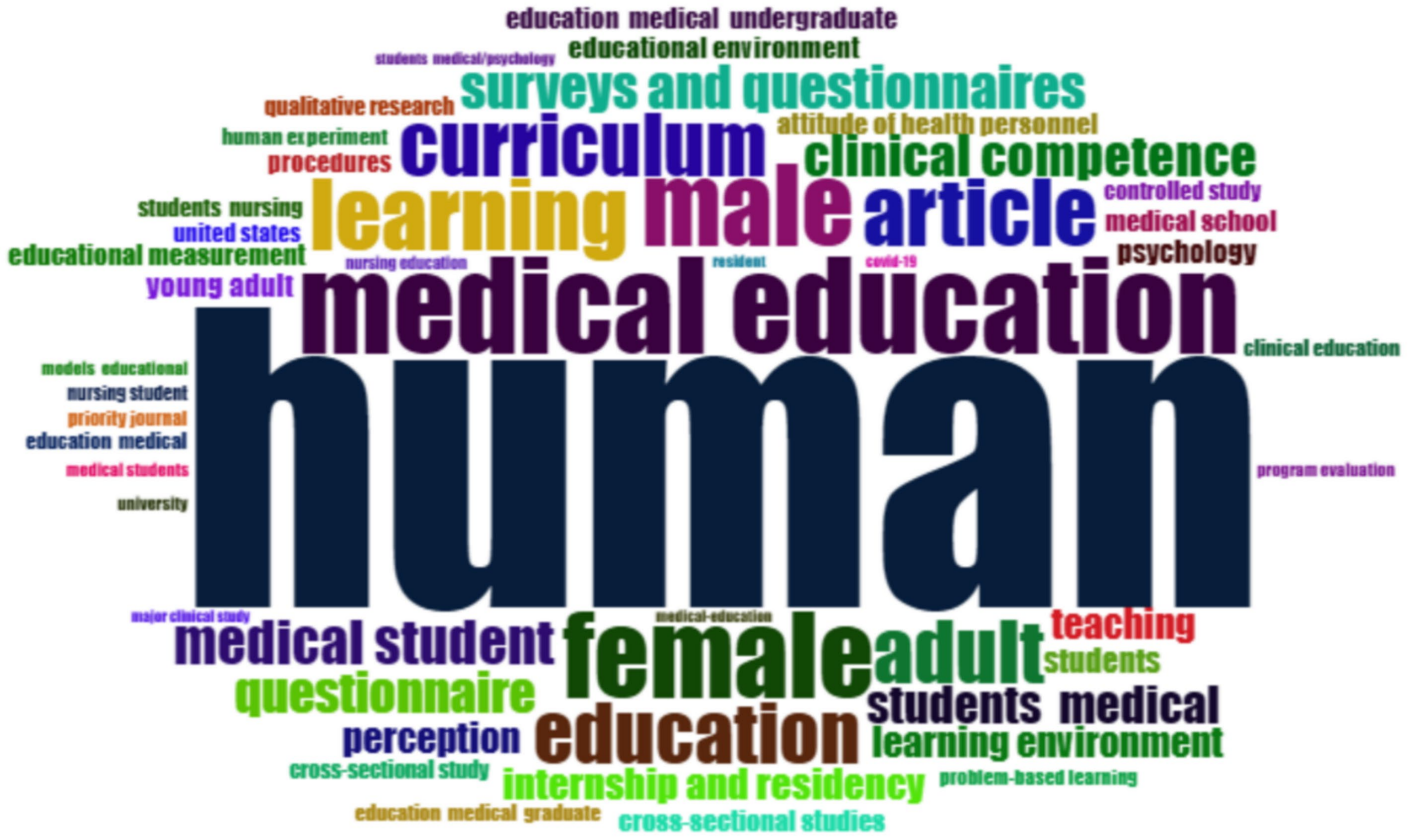
Word cloud of the key terms in medical school learning environment (MSLE).

The word cloud visualization (Figure 11) illustrates the most frequently occurring author-supplied keywords in the bibliometric dataset, providing a descriptive overview of the dominant themes in the literature on medical school learning environments. Several highly frequent terms (e.g., “human,” “male,” and “female”) reflect common descriptive and indexing practices within bibliographic records rather than standalone educational themes and were therefore interpreted in context. Beyond these generic descriptors, the word cloud highlights a strong concentration of conceptually meaningful terms directly related to medical education, including “learning,” “curriculum,” “medical education,” “education,” and “medical student.” Keywords associated with learning outcomes and assessment such as “clinical competence,” “surveys and questionnaires,” “educational measurement,” and “program evaluation” also appear prominently, underscoring the field’s emphasis on evaluation and measurement of educational environments. In addition, pedagogical and contextual terms including “teaching,” “perception,” “learning environment,” and “problem-based learning” are consistently represented (Supplementary material 21). Collectively, these patterns illustrate the breadth of topics examined in MSLE research and provide a descriptive overview of recurrent educational concepts in the field.

**Figure 12:**
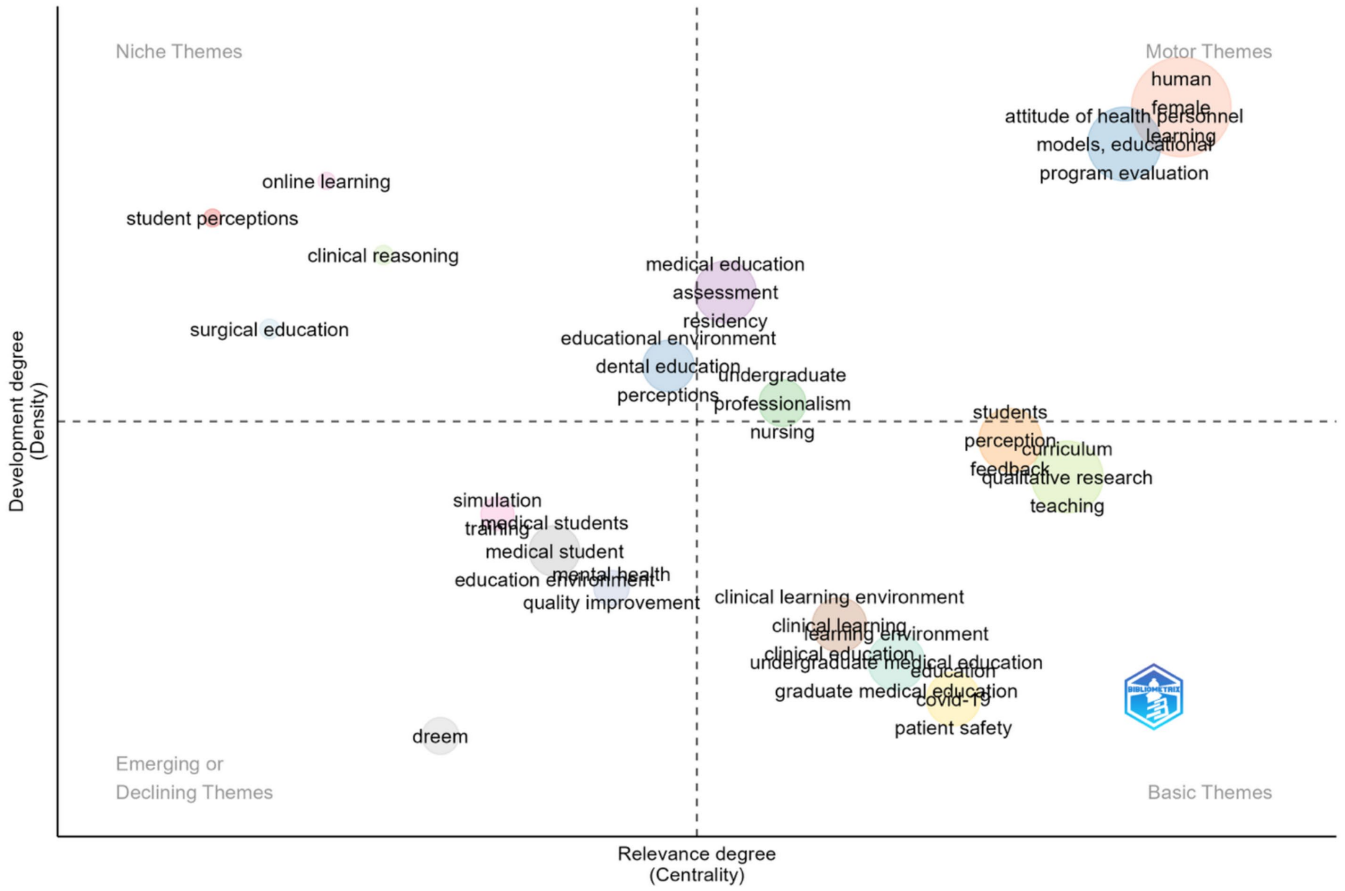
Thematic map.

A thematic map based on keyword co-occurrence categorizes research topics according to their relevance (centrality) and degree of development (density) (Figure 12). Several highly central terms appear in the motor-theme quadrant, reflecting frequently used author keywords and indexing descriptors; these were interpreted in context rather than as standalone educational constructs. Among the conceptually meaningful motor themes, professionalism, program evaluation, and attitudes of health personnel emerge as well-developed and influential areas within MSLE research. Basic themes characterized by high relevance but lower developmental density include the clinical learning environment, undergraduate medical education, curriculum, teaching, and patient safety, indicating foundational domains that remain central to the field and offer scope for further development. Niche themes, which are well developed but more specialized, include online learning, student perceptions, clinical reasoning, and surgical education, reflecting focused areas of inquiry with limited cross-thematic integration. Emerging or potentially declining themes, such as simulation, quality improvement, and education environment, suggest areas where research activity may be evolving or where future integration into broader MSLE frameworks is needed. Collectively, the thematic map illustrates the structural organization of MSLE research and highlights variation in thematic maturity and influence across the field.

### Most widely used learning environment assessment tool

Analysis of the included publications revealed that the Dundee Ready Educational Environment Measure (DREEM) was the most frequently employed instrument for assessing the learning environment. Developed by Roff et al. (15), DREEM is a 50-item questionnaire encompassing five dimensions: students’ perceptions of learning, teachers, academic self-perceptions, atmosphere, and social self-perceptions.

## Discussion

In this study, we conducted a comprehensive bibliometric analysis of the literature on MSLE published between 2000 and mid-2025. Publication trends were examined to track the annual growth of research output and average citation patterns, while geographical distribution highlighted the leading countries and regions contributing to the field. Source analysis provided insights into the journals most actively publishing MSLE research and their relative impact, whereas author- and institution-level assessments identified the most influential contributors and collaborative networks shaping the discourse. At the country level, patterns of corresponding authorship and international collaboration were explored to reveal the global research landscape. Highly cited publications were analyzed to showcase the seminal works that have most strongly influenced the field. Finally, keyword co-occurrence and thematic mapping were used to identify dominant research themes, emerging areas, and potential directions for future inquiry. Together, these bibliometric parameters offer a holistic overview of the structure, development, and evolving trends in MSLE research. As a bibliometric study, these findings should be interpreted as reflecting trends, emphases, and gaps in MSLE research, rather than direct evidence of the quality or characteristics of learning environments within medical schools.

### Annual trends in scientific production

The steady increase in annual publications from 2000 onwards reflects a sustained expansion of scholarly interest in MSLE (25). Between 2000 and 2005, outputs remained modest, but the sharp rise after 2010 suggests that the concept of LE became more firmly embedded in medical education discourse, coinciding with growing recognition of its impact on student outcomes, professional identity formation, and accreditation requirements. The peak in 2024 with 326 publications signals not only the field’s ongoing relevance but also its recognition as a central determinant of medical education quality (7, 26).

The citation trajectory provides further insights into these dynamics. A pronounced peak in 2020 with average citations exceeding 2.5 per article likely reflects the rapid uptake of studies published shortly before and during the COVID-19 pandemic, when shifts to online learning, virtual environments, and student wellbeing concerns brought renewed attention to the learning environment (27, 28). This surge aligns with a broader pivot in medical education research toward digital pedagogy and psychological support frameworks. The subsequent decline in 2024–2025 citation averages is consistent with bibliometric lag effects, as newer articles typically require more time to accumulate recognition and impact within the field.

### Geographical distribution of research output

Country-level analysis reveals a concentration of research in high-income countries, with the United States dominating the field (*n* = 2,364), followed by Australia, Canada, China, and United Kingdom. This pattern mirrors bibliometric trends in medical education more broadly, where U.S. and Commonwealth nations have historically shaped scholarship and global standards (29). China’s rapid rise in later years reflects an important shift, aligning with its broader strategy to internationalize higher education and medical research (30). Similarly, Saudi Arabia’s strong performance within the GCC underscores the region’s increasing investment in medical education reforms and accreditation (31). The UAE, though smaller in output, demonstrates growing contributions, consistent with institutional priorities to achieve international accreditation and strengthen educational research (32, 33). Despite these successes, notable geographic disparities persist. Low- and middle-income countries (LMICs) outside the GCC remain underrepresented, raising questions about the global transferability of MSLE findings. The limited visibility of African, South American, and Southeast Asian institutions suggests a need for more inclusive research networks to address context-specific challenges in diverse learning environments (34).

### Analysis of journals and sources

The analysis of journals publishing in the field of MSLE highlights several important trends that reflect both the evolution of the field and the dissemination of its knowledge. Our findings indicate that BMC Medical Education has emerged as the most prolific platform, publishing nearly 300 articles on the topic since 2005. Its open-access model and broad readership likely contribute to its dominant role in disseminating research on learning environments, particularly in recent years when global access and visibility have become increasingly important for academic exchange (35). Established journals such as Medical Teacher, Academic Medicine, and Medical Education remain central to the field, not only in terms of productivity but also in scientific impact, as reflected by their high h-indices. These outlets have historically been considered the “core journals” of medical education research and continue to shape discourse around learning environments, assessment, curriculum innovation, and faculty development (29). The emergence of nursing-focused journals (Nurse Education Today, Nurse Education in Practice) and specialty outlets (Journal of Surgical Education, Teaching and Learning in Medicine) among the most productive sources underscores the interdisciplinary reach of LE research. This trend reflects the broader recognition that supportive and effective LEs are vital not only for medical students but also across the spectrum of health professions education, including nursing, dentistry, and surgical training (36–38).

Altogether, these patterns suggest that LE research is now firmly embedded within the broader medical education literature, with both traditional high-impact journals and newer, open-access outlets playing complementary roles. Established journals continue to anchor scholarly discourse, while open-access platforms accelerate knowledge sharing across diverse regions and professional groups (39, 40). This dual structure has likely contributed to the recent surge in output, supporting the field’s relevance and reinforcing its central role in shaping educational practice and policy worldwide.

### Authors analysis

Our results show that research on MSLE is shaped by a cluster of highly productive scholars who continue to anchor the field, complemented by newer contributors who are steadily expanding its scope. This distribution reflects a common pattern in medical education research, where a handful of pioneers lay the conceptual foundations and their networks drive sustained productivity and impact, while emerging voices bring diversification and innovation to the discourse. At the same time, the observed author collaboration patterns indicate that although certain research groups demonstrate strong internal cohesion, cross-group and international connectivity remain limited, resulting in relatively fragmented scholarly networks. Such structural fragmentation has been noted in prior bibliometric analyses of medical education and related health professions research, where low network density was found to restrict the cross-fertilization of ideas and limit the development of larger, interdisciplinary collaborations (41, 42). The coexistence of long-standing leaders and newer contributors thus highlights both the maturity of the field and the need to foster stronger collaborative ties across regions and disciplines to accelerate knowledge integration and address global challenges in the domain of MSLE (43, 44).

### Institutional contributions

Our results highlight the dominance of North American institutions as leading contributors to research on MSLE. This pattern reflects their longstanding investment in educational research infrastructure, availability of dedicated centers for medical education, and strong funding ecosystems. Canadian institutions also feature prominently, underscoring the country’s reputation for advancing competency-based medical education and integrating educational scholarship into faculty promotion pathways. Together, the U.S. and Canada account for a large proportion of global output, consistent with previous bibliometric studies showing that North America remains the most productive hub in medical education research (29, 43, 45). Beyond North America, notable contributions from European and Asia-Pacific institutions such as Maastricht University, Monash University, and the University of Otago demonstrate the growing diversification of global leadership in this field. Mid-to-high output institutions, including Johns Hopkins, Baylor College of Medicine, and Northwestern University, also play important roles as research nodes, supporting both domestic and international collaborations. However, the limited representation of institutions from Africa, Latin America, and much of Asia highlights persistent geographic inequities in medical education research (34). Expanding institutional partnerships in these underrepresented regions will be critical to fostering more globally inclusive perspectives and contextually relevant approaches to improving MSLE.

### Country-level collaboration and impact

The country-level analysis underscores the United States’ central role not only in productivity but also in shaping global collaboration networks in medical education research. While U.S. publications dominate numerically, their low proportion of international co-authorship (3.9%) suggests a primarily domestic focus, contrasting with countries such as the United Kingdom, Canada, and the Netherlands, where higher rates of multi-country publications indicate more active global engagement (45). Emerging contributors from the Middle East (46), particularly Saudi Arabia, demonstrate notable international integration relative to their output, with 15.2% of publications involving cross-border collaboration (47, 48). Their growing visibility in terms of citations and average impact reflects broader regional efforts to strengthen medical education research and forge global partnerships. Similarly, countries in Asia such as China, Japan, and Singapore are increasingly connected within the global collaboration network, signaling diversification of scholarly influence beyond traditional Western hubs (49).

### Highly cited publications

The analysis of highly cited publications reveals both the enduring influence of foundational contributions and the rapid rise of contemporary works in MSLE. Earlier studies, which focused on outcome-based frameworks, simulation, and evidence-based teaching methods, continue to attract sustained citations, reflecting their role in shaping core pedagogical principles. Their long-term impact illustrates how seminal frameworks, and systematic evaluations provided the conceptual backbone for much of the subsequent research in the field. In contrast, more recent publications demonstrate accelerated citation growth, particularly those addressing digital transformation, online learning, and virtual education. The surge in annual citation rates highlights how the field has responded swiftly to global disruptions and technological shifts, with research outputs rapidly entering mainstream scholarly and institutional discourse. This pattern reflects the increasing prioritization of adaptable, technology-enabled educational models capable of addressing emergent challenges.

### Thematic evolution of research

The thematic evolution shows that research on MSLE continues to focus on core issues such as curriculum, clinical competence, and program evaluation. This reflects the wider trend toward competency-based medical education, where developing and measuring learner outcomes is a priority (50). The frequent appearance of terms like perception and learning environment also highlights how strongly student experiences shape engagement and learning, as supported by international studies using tools such as the DREEM survey (15, 51). The thematic map adds further depth by illustrating how topics are positioned within the field. Professionalism and program evaluation emerge as central, well-developed themes, while areas such as curriculum, patient safety, and the clinical learning environment remain highly relevant but show comparatively lower thematic density, suggesting scope for further conceptual integration. More specific areas such as online learning and surgical education have grown rapidly, particularly during the COVID-19 pandemic (52), though they remain somewhat separate from broader MSLE research. Interestingly, themes like simulation and quality improvement are placed as emerging or potentially declining, indicating that future research may benefit from integrating these approaches more explicitly into system-level and curricular frameworks (53). Overall, the results suggest that MSLE scholarship remains largely anchored in perception- and measurement-based evaluation of educational environments, with growing attention to clinical and digital learning contexts; however, comparatively fewer studies examine the effectiveness of institutional interventions or longitudinal change, highlighting important priorities for future research.

### Most widely used learning environment assessment tool

The predominance of DREEM in the analyzed literature underscores its position as the most established and globally validated instrument for evaluating the learning environment in medical education (15, 54). Its frequent appearance in highly cited articles reflects both its methodological robustness and its widespread acceptance as a benchmark for LE assessment (20, 55, 56). The DREEM’s versatility being culturally non-specific and adaptable across diverse educational contexts explains its sustained prominence over nearly three decades (8, 57, 58). This trend suggests that DREEM remains a reliable tool for comparative and cross-institutional studies; however, further adaptation and validation may be warranted to align it with emerging learning paradigms, including hybrid and technology-enhanced environments.

## Conclusion

This bibliometric analysis highlights the remarkable expansion of research on MSLE over the past two decades, underscoring its growing recognition within medical education scholarship. Our findings reveal clear upward trends in publication output, increased contributions from diverse regions, and the emergence of both pioneering and new voices advancing the field. Leading journals and institutions continue to anchor scholarship, while evolving themes such as program evaluation, digital pedagogy, and student wellbeing reflect the shifting scholarly priorities in medical education worldwide. Overall, these patterns indicate that the learning environment has become a central focus of medical education research, closely linked to questions of educational quality, professional development, and learner outcomes.

### Limitations and future direction

Despite its comprehensive scope, our study has certain limitations. First, although we searched three major databases (PubMed, Scopus, and Web of Science) to maximize coverage, some relevant publications may still have been missed due to indexing inconsistencies or variations in terminology. Second, records for 2025 represent a partial year because the search was conducted on July 17, 2025. As a result, publication and citation trends for 2025 may appear artificially lower than complete years and should be interpreted with this limitation in mind. Third, our search was restricted to English-language publications. This may bias the results toward English-language journals and countries with high English publication output, potentially contributing to the prominence of countries such as the United States, Australia, and Canada in our dataset. Consequently, MSLE scholarship published in other languages may be underrepresented, and findings regarding global distribution should be interpreted accordingly. Finally, analyses of institutional and country affiliations can be affected by variations in how these are reported, which may lead to fragmentation of institutional outputs.

Looking ahead, future research would benefit from longitudinal, multi-institutional investigations that examine how medical school learning environments are studied over time and how research in this area responds to curricular reform, technological innovation, and shifting student needs. Such approaches could better capture the dynamic and context-dependent nature of educational ecosystems and allow evaluation of the effectiveness of institutional interventions. Furthermore, sustained international collaboration and inclusion of underrepresented regions will be critical to building a more global, contextually relevant evidence base that can shape policy and practice in medical education.

## Data Availability

The original contributions presented in the study are included in the article/Supplementary material, further inquiries can be directed to the corresponding author.
