# Supplementary material for: A bibliometric analysis of research on the learning environment in medical schools: trends, gaps, and global perspectives
Source: Front Med (Lausanne). 2026 Feb 3;13:1760995. doi: 10.3389/fmed.2026.1760995 (PMC12909209; doi:10.3389/fmed.2026.1760995)
Supplement: Supplementary file 1 [file Data_Sheet_1.pdf]

# *Supplementary Material*

| **Supplementary Material 1.** A detailed description of the search terms and strategy. | | | |
|---|---|---|---|
| **Source and coverage** | **Search string** | **Results** | **notes** |
| **PubMed (NLM)**<br>**Coverage:**<br>From database inception-2025-07-17 | **(("Learning Environment"[Title/Abstract] OR "Clinical Learning Environment"[Title/Abstract] OR "Learning Laboratory"[Title/Abstract] OR "Environment Design"[Title/Abstract] OR "Educational Environment"[Title/Abstract] OR "Academic Environment"[Title/Abstract] OR "Clinical Learning Environment"[Title/Abstract] OR "Teaching Environment"[Title/Abstract]) AND ("Medical Education"[Title/Abstract] OR "education, medical"[MeSH Terms] OR "Teaching"[MeSH Terms] OR "schools, medical"[MeSH Terms] OR "Medical Schools"[Title/Abstract] OR "education undergraduate medical"[Title/Abstract] OR "medical education undergraduate"[Title/Abstract] OR "Undergraduate Medical Education"[Title/Abstract] OR "faculty medical"[Title/Abstract] OR "Medical College"[Title/Abstract] OR "Medical Faculty"[Title/Abstract] OR "schools medical"[Title/Abstract] OR "education medical"[Title/Abstract] OR "education medical graduate"[Title/Abstract] OR "education medical undergraduate"[Title/Abstract] OR "education medical"[Title/Abstract] OR "Medical instruction"[Title/Abstract] OR "Medical Teaching"[Title/Abstract] OR "Medical Training"[Title/Abstract] OR "training medical"[Title/Abstract] OR "Teaching"[Title/Abstract] OR "Medical Students"[Title/Abstract] OR "Student Perceptions"[Title/Abstract] OR "Medical curriculum"[Title/Abstract])) AND ((bibliography[Filter] OR casereports[Filter] OR clinicalconference[Filter] OR clinicalstudy[Filter] OR clinicaltrial[Filter] OR clinicaltrialprotocol[Filter] OR clinicaltrialphasei[Filter] OR clinicaltrialphaseii[Filter] OR clinicaltrialphaseiii[Filter] OR clinicaltrialphaseiv[Filter] OR comparativestudy[Filter] OR consensusdevelopmentconferencenih[Filter] OR controlledclinicaltrial[Filter] OR evaluationstudy[Filter] OR meta-analysis[Filter] OR multicenterstudy[Filter] OR observationalstudy[Filter] OR practiceguideline[Filter] OR randomizedcontrolledtrial[Filter] OR researchsupportnihextramural[Filter] OR researchsupportnihintramural[Filter] OR researchsupportnonusgovt[Filter] OR review[Filter] OR scientificintegrityreview[Filter] OR scopingreview[Filter] OR systematicreview[Filter] OR technicalreport[Filter] OR validationstudy[Filter]) AND (humans[Filter]) AND (english[Filter]) AND (2000:2025[pdat]))** | **1481** | All search terms are searched in the field: [Title/Abstract] and in MeSH (when available). filters or English language, articles and reviews<br>**Years 2000-2025** |
| **Scopus (Elsevier)**<br>**Coverage:**<br>From database inception-2025-07-17 | ( TITLE-ABS-KEY ( "Clinical Learning" ) OR TITLE-ABS-KEY ( "Environment, Learning" ) OR TITLE-ABS-KEY ( "Learning Laboratory" ) OR TITLE-ABS-KEY ( "Environment, Learning" ) OR TITLE-ABS-KEY ( "Environment Design" ) OR TITLE-ABS-KEY ( "Educational Environment" ) OR TITLE-ABS-KEY ( "Academic Environment" ) OR TITLE-ABS-KEY ( "Clinical Learning Environment" ) OR TITLE-ABS-KEY ( "Teaching Environment" ) ) AND ( TITLE-ABS-KEY ( "Medical School" ) OR TITLE-ABS-KEY ( "Medical Schools" ) OR TITLE-ABS-KEY ( "School, Medical" ) OR TITLE-ABS-KEY ( "Medical Education" ) OR TITLE-ABS-KEY ( "Education, Undergraduate Medical" ) OR TITLE-ABS-KEY ( "Medical Education, Undergraduate" ) OR TITLE-ABS-KEY ( "Undergraduate Medical Education" ) OR TITLE-ABS-KEY ( "Faculty, Medical" ) OR TITLE-ABS-KEY ( "Medical College" ) OR TITLE-ABS-KEY ( "Schools, Medical" ) OR TITLE-ABS-KEY ( "Education Centers" ) OR TITLE-ABS-KEY ( "Education, Medical" ) OR TITLE-ABS-KEY ( "Education, Medical, graduate" ) OR TITLE-ABS-KEY ( "Education, Medical, undergraduate" ) OR TITLE-ABS-KEY ( "Medical instruction" ) OR TITLE-ABS-KEY ( "Medical Teaching" ) OR TITLE-ABS-KEY ( "Medical Training" ) OR TITLE-ABS-KEY ( "Training, Medical" ) OR TITLE-ABS-KEY ( "Medical Students" ) OR TITLE-ABS-KEY ( "Student Perceptions" ) OR TITLE-ABS-KEY ( "Medical curriculum" ) ) AND PUBYEAR > 1999 AND PUBYEAR < 2026 AND ( LIMIT-TO ( DOCTYPE , "ar" ) OR LIMIT-TO ( DOCTYPE , "re" ) OR LIMIT-TO ( DOCTYPE , "cp" ) ) AND ( LIMIT-TO ( LANGUAGE , "English" ) ) AND ( LIMIT-TO ( EXACTKEYWORD , "Medical Education" ) OR LIMIT-TO ( EXACTKEYWORD , "Medical Student" ) OR LIMIT-TO ( EXACTKEYWORD , "Learning Environment" ) OR LIMIT-TO ( EXACTKEYWORD , "Students, Medical" ) OR LIMIT-TO ( EXACTKEYWORD , "Medical School" ) OR LIMIT- | **2115** | All search terms are searched in the fields: “title”, “abstract” and “keywords” (here marked with “TITLE-ABS-KEY”) filters or limitations English language, articles and reviews<br>**Years 2000-2025** |

| | | | |
|---|---|---|---|
| | TO ( EXACTKEYWORD , "Education, Medical, Undergraduate" ) OR LIMIT-TO ( EXACTKEYWORD , "Education, Medical" ) OR LIMIT-TO ( EXACTKEYWORD , "Educational Environment" ) OR LIMIT-TO ( EXACTKEYWORD , "Medical Students" ) OR LIMIT-TO ( EXACTKEYWORD , "Schools, Medical" ) OR LIMIT-TO ( EXACTKEYWORD , "Program Evaluation" ) OR LIMIT-TO ( EXACTKEYWORD , "Clinical Learning Environment" ) OR LIMIT-TO ( EXACTKEYWORD , "Learning" ) OR LIMIT-TO ( EXACTKEYWORD , "Questionnaire" ) OR LIMIT-TO ( EXACTKEYWORD , "Curriculum" ) OR LIMIT-TO ( EXACTKEYWORD , "Surveys And Questionnaires" ) OR LIMIT-TO ( EXACTKEYWORD , "Perception" ) OR LIMIT-TO ( EXACTKEYWORD , "Cross-sectional Study" ) OR LIMIT-TO ( EXACTKEYWORD , "Faculty, Medical" ) OR LIMIT-TO ( EXACTKEYWORD , "Educational Measurement" ) OR LIMIT-TO ( EXACTKEYWORD , "Qualitative Research" ) OR LIMIT-TO ( EXACTKEYWORD , "Self Concept" ) OR LIMIT-TO ( EXACTKEYWORD , "Clinical Clerkship" ) OR LIMIT-TO ( EXACTKEYWORD , "Accreditation" ) OR LIMIT-TO ( EXACTKEYWORD , "Student Attitude" ) OR LIMIT-TO ( EXACTKEYWORD , "Dreem" ) ) AND ( LIMIT-TO ( SUBJAREA , "MEDI" ) OR LIMIT-TO ( SUBJAREA , "SOCI" ) OR LIMIT-TO ( SUBJAREA , "HEAL" ) OR LIMIT-TO ( SUBJAREA , "ENVI" ) ) | | |
| **Web of Science-Core Collection (Clarivate)**<br>**Coverage:**<br>From database inception-2025-07-17 | **#1 (((((((TS=("Clinical Learning ")) OR TS=("Environment, Learning")) OR TS=("Learning Laboratory")) OR TS=("Environment, Learning")) OR TS=("Environment Design")) OR TS=("Educational Environment")) OR TS=("Academic Environment")) OR TS=("Clinical Learning Environment")) OR TS=("Teaching Environment")**<br><br>**#2 (((((((((((((((((TS=("Medical School")) OR TS=("Medical Schools")) OR TS=("School, Medical")) OR TS=("Medical Education")) OR TS=("Education, Undergraduate Medical")) OR TS=("Medical Education, Undergraduate")) OR TS=("Undergraduate Medical Education")) OR TS=("Faculty, Medical")) OR TS=("Medical College")) OR TS=("Medical Faculty")) OR TS=("Schools, Medical")) OR TS=("Area Health Education Centres")) OR TS=("Education, Medical")) OR TS=("Education, Medical, graduate")) OR TS=("Education, Medical, undergraduate")) OR TS=("Education, Medical")) OR TS=("Medical instruction")) OR TS=("Medical Teaching")) OR TS=("Medical Training")) OR TS=("Training, Medical")**<br><br>**#1 AND #2** and **2000** or **2001** or **2002** or **2003** or **2004** or **2005** or **2006** or **2007** or **2008**or **2009** or **2010** or **2012** or **2011** or **2025** or **2024** or **2023** or **2022** or **2021** or **2020** or **2019** or **2018** or **2017** or **2016** or **2015** or **2014** or **2013** (Publication Years) and **Article**or **Review Article** (Document Types) and **English** (Languages) and **Education Educational Research** or **Health Care Sciences Services** or **General Internal Medicine** or **Public Environmental Occupational Health** or **Medical Informatics** or **Psychiatry** or **Psychology** or **Biomedical Social Sciences** (Research Areas)<br><br>https://www.webofscience.com/wos/woscc/summary/be702bb1-7c73-416e-b718-1ddcc4f2ad34-016f0f7584/relevance/1 | **864** | All search terms are searched in the field: “Topic” (including title, abstract and author supplied keywords, here marked with “TOPIC”). filters or limitations English language, articles and reviews<br>**Years 2000-2025** |
| **Total no. of references identified** | | **4460** | |
| **Total no. unique references identified after automatic de-duplication in Bibliometrix** | | **3458** | |
| **Duplicates identified** | | **1002** | |

**Supplementary Material 2.** Annual scientific production

| Year | Articles |
|---|---|
| 2000 | 29 |
| 2001 | 35 |
| 2002 | 29 |
| 2003 | 36 |

| | |
|---|---|
| 2004 | 61 |
| 2005 | 66 |
| 2006 | 94 |
| 2007 | 71 |
| 2008 | 72 |
| 2009 | 94 |
| 2010 | 91 |
| 2011 | 99 |
| 2012 | 118 |
| 2013 | 111 |
| 2014 | 148 |
| 2015 | 167 |
| 2016 | 168 |
| 2017 | 140 |
| 2018 | 158 |
| 2019 | 203 |
| 2020 | 204 |
| 2021 | 255 |
| 2022 | 278 |
| 2023 | 237 |
| 2024 | 326 |
| 2025 | 168 |

**Supplementary Material 3.** Annual total citation per year

| Year | MeanTCperArt | N | MeanTCperYear | Citable years |
|---|---|---|---|---|
| 2000 | 30.55 | 29 | 1.18 | 26 |
| 2001 | 28.54 | 35 | 1.14 | 25 |
| 2002 | 13.17 | 29 | 0.55 | 24 |
| 2003 | 26.39 | 36 | 1.15 | 23 |
| 2004 | 36.00 | 61 | 1.64 | 22 |
| 2005 | 33.61 | 66 | 1.60 | 21 |
| 2006 | 31.43 | 94 | 1.57 | 20 |
| 2007 | 31.76 | 71 | 1.67 | 19 |
| 2008 | 18.06 | 72 | 1.00 | 18 |
| 2009 | 27.38 | 94 | 1.61 | 17 |
| 2010 | 20.93 | 91 | 1.31 | 16 |
| 2011 | 10.44 | 99 | 0.70 | 15 |
| 2012 | 20.33 | 118 | 1.45 | 14 |
| 2013 | 19.06 | 111 | 1.47 | 13 |
| 2014 | 14.75 | 148 | 1.23 | 12 |
| 2015 | 16.49 | 167 | 1.50 | 11 |
| 2016 | 13.61 | 168 | 1.36 | 10 |
| 2017 | 13.84 | 140 | 1.54 | 9 |
| 2018 | 13.23 | 158 | 1.65 | 8 |
| 2019 | 10.60 | 203 | 1.51 | 7 |
| 2020 | 15.42 | 204 | 2.57 | 6 |
| 2021 | 9.44 | 255 | 1.89 | 5 |
| 2022 | 4.97 | 278 | 1.24 | 4 |
| 2023 | 2.95 | 237 | 0.98 | 3 |
| 2024 | 1.40 | 326 | 0.70 | 2 |
| 2025 | 0.18 | 168 | 0.18 | 1 |

Abbreviations: MeanTCperArt, mean total citations per article; N, number; MeanTCperYear, Mean total citation per year.

| Supplementary Material 4. Country production | | |
|---|---|---|
| No. | Country | Frequency |
| 1 | USA | 2364 |
| 2 | AUSTRALIA | 569 |
| 3 | CANADA | 550 |
| 4 | CHINA | 503 |
| 5 | UK | 372 |
| 6 | NETHERLANDS | 281 |
| 7 | SINGAPORE | 189 |
| 8 | BRAZIL | 172 |
| 9 | PAKISTAN | 167 |
| 10 | IRAN | 141 |
| 11 | JAPAN | 128 |
| 12 | SAUDI ARABIA | 128 |
| 13 | GERMANY | 127 |
| 14 | IRELAND | 116 |
| 15 | INDIA | 113 |

| Supplementary Material 5. GCC countries production | | |
|---|---|---|
| No. | Country | Frequency |
| 1 | SAUDI ARABIA | 128 |
| 2 | UNITED ARAB EMIRATES | 52 |
| 3 | QATAR | 21 |
| 4 | OMAN | 11 |
| 5 | KUWAIT | 8 |
| 6 | BAHRAIN | 2 |
| Abbreviations: GCC, Gulf Cooperation Council. | | |

**Supplementary Material 6. GCC country production**

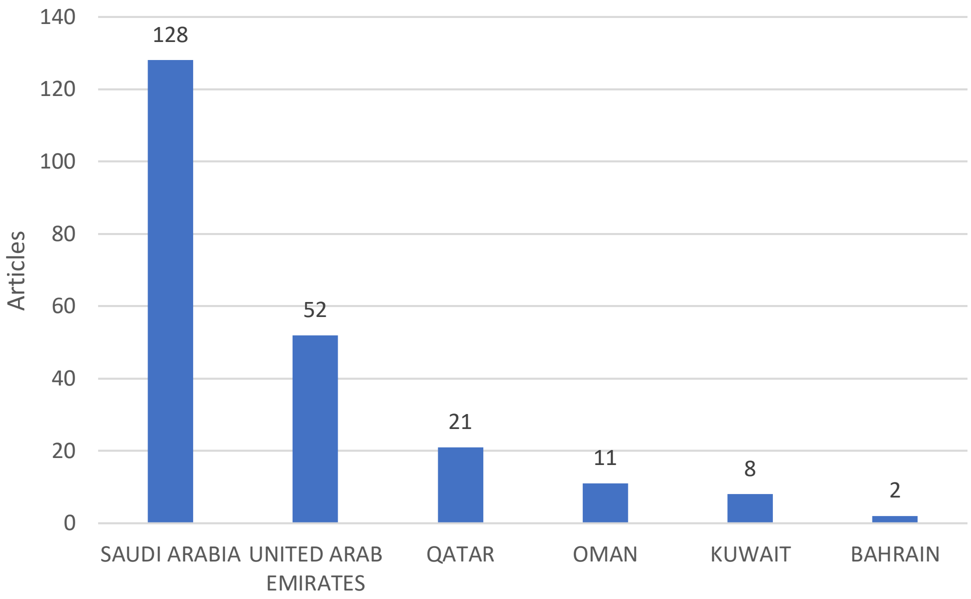


Abbreviations: GCC, Gulf Cooperation Council.

**Supplementary Material 7.** Most cited countries

| No. | Country | Total Count | Average Article Citations |
|---|---|---|---|
| 1 | USA | 12060 | 18.80 |
| 2 | UNITED KINGDOM | 6300 | 35.80 |
| 3 | CANADA | 3708 | 30.90 |
| 4 | AUSTRALIA | 3064 | 22.40 |
| 5 | NETHERLANDS | 1325 | 18.70 |
| 6 | NEW ZEALAND | 1102 | 28.30 |
| 7 | SAUDI ARABIA | 986 | 14.90 |
| 8 | IRELAND | 757 | 22.30 |
| 9 | IRAN | 691 | 11.10 |
| 10 | BRAZIL | 650 | 22.40 |
| 11 | CHINA | 646 | 9.20 |
| 12 | SWEDEN | 619 | 20.60 |
| 13 | INDIA | 610 | 12.20 |
| 14 | KOREA | 590 | 19.70 |
| 15 | ISRAEL | 506 | 42.20 |

**Supplementary Material 8. Most cited countries**

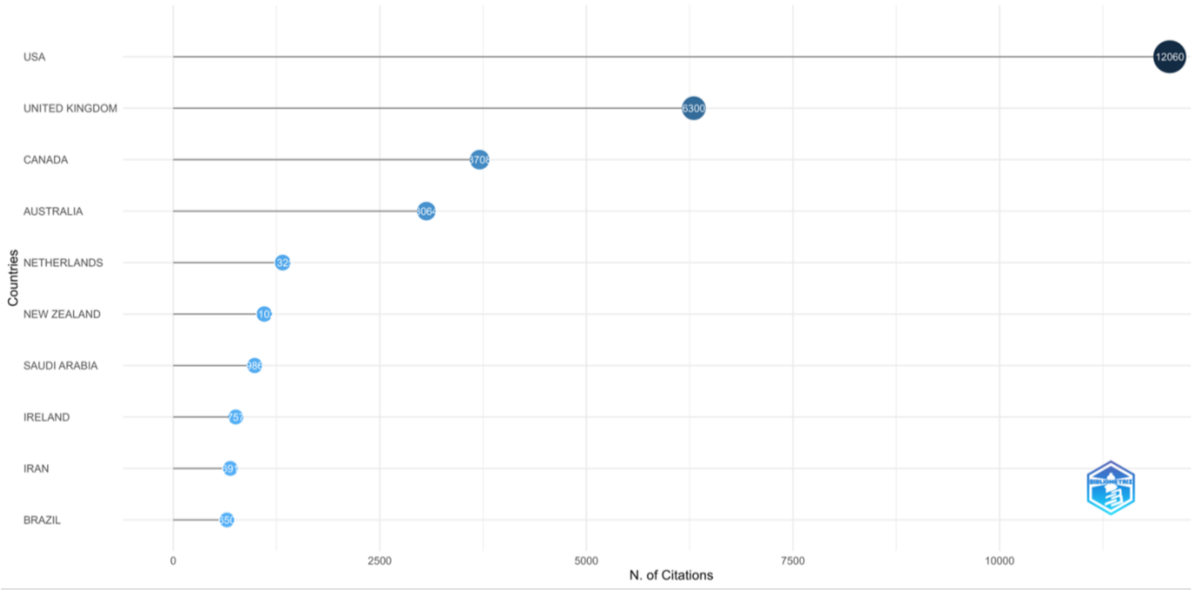


| **Supplementary Material 9.** Most Relevant Countries by Corresponding Author | | | | | | |
|---|---|---|---|---|---|---|
| **No.** | **Country** | **Articles** | **Articles %** | **SCP** | **MCP** | **MCP %** |
| 1 | USA | 643 | 18.6 | 618 | 25 | 3.9 |
| 2 | UNITED KINGDOM | 176 | 5.1 | 160 | 16 | 9.1 |
| 3 | AUSTRALIA | 137 | 4 | 129 | 8 | 5.8 |
| 4 | CANADA | 120 | 3.5 | 110 | 10 | 8.3 |
| 5 | NETHERLANDS | 71 | 2.1 | 66 | 5 | 7 |
| 6 | CHINA | 70 | 2 | 65 | 5 | 7.1 |
| 7 | PAKISTAN | 66 | 1.9 | 61 | 5 | 7.6 |
| 8 | SAUDI ARABIA | 66 | 1.9 | 56 | 10 | 15.2 |
| 9 | IRAN | 62 | 1.8 | 61 | 1 | 1.6 |
| 10 | INDIA | 50 | 1.4 | 49 | 1 | 2 |
| 11 | NEW ZEALAND | 39 | 1.1 | 37 | 2 | 5.1 |
| 12 | IRELAND | 34 | 1 | 30 | 4 | 11.8 |
| 13 | SOUTH AFRICA | 33 | 1 | 33 | 0 | 0 |
| 14 | MALAYSIA | 32 | 0.9 | 30 | 2 | 6.3 |
| 15 | KOREA | 30 | 0.9 | 28 | 2 | 6.7 |
| Abbreviations: SCP, Single Country Publications; MCP, Multiple Country Publications. | | | | | | |

**Supplementary Material 10.** Most relevant sources

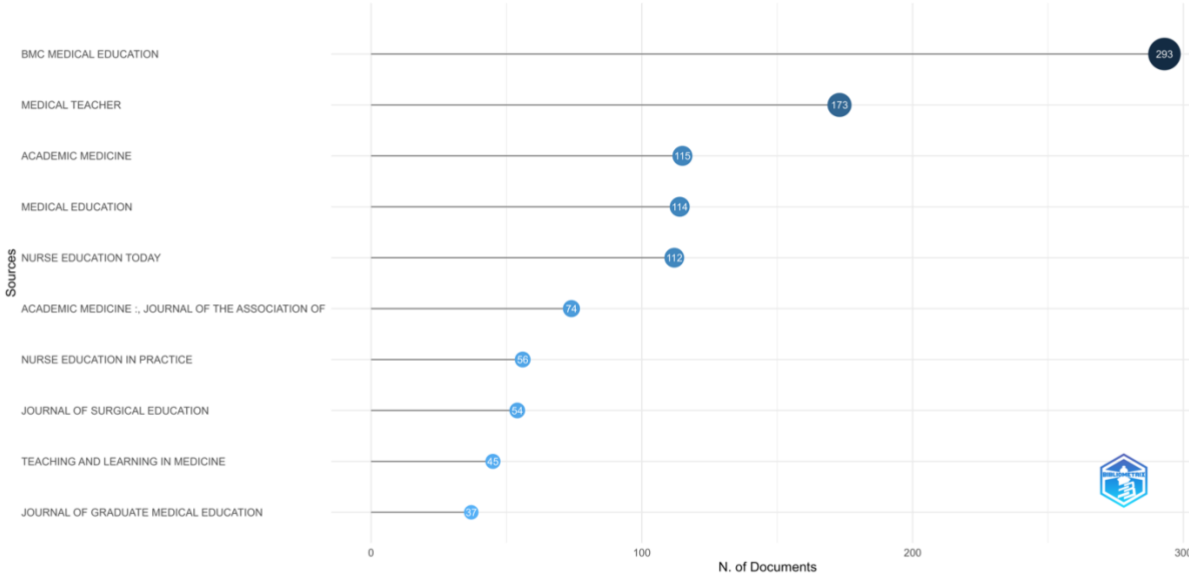


**Supplementary Material 11.** Sources local impact

| No. | Source | h-index | g-index | m-index | TC | NP | PY start |
|---|---|---|---|---|---|---|---|
| 1 | MEDICAL TEACHER | 42 | 78 | 1.61538462 | 6609 | 173 | 2000 |
| 2 | ACADEMIC MEDICINE | 35 | 62 | 1.34615385 | 4264 | 115 | 2000 |
| 3 | MEDICAL EDUCATION | 33 | 55 | 1.26923077 | 3078 | 114 | 2000 |
| 4 | BMC MEDICAL EDUCATION | 31 | 56 | 1.47619048 | 3892 | 293 | 2005 |
| 5 | NURSE EDUCATION TODAY | 26 | 47 | 1.04 | 2284 | 112 | 2001 |
| 6 | ADVANCES IN HEALTH SCIENCES EDUCATION | 15 | 26 | 0.78947368 | 685 | 28 | 2007 |
| 7 | NURSE EDUCATION IN PRACTICE | 15 | 28 | 0.68181818 | 793 | 56 | 2004 |
| 8 | MEDICAL EDUCATION ONLINE | 13 | 21 | 0.8125 | 485 | 36 | 2010 |
| 9 | EUROPEAN JOURNAL OF DENTAL EDUCATION | 12 | 19 | 0.70588235 | 389 | 22 | 2009 |
| 10 | JOURNAL OF GENERAL INTERNAL MEDICINE | 12 | 29 | 0.52173913 | 884 | 30 | 2003 |

Abbreviations: TC, total citations; NP, number of publications; PY, publication year.

**Supplementary Material 12.** Sources local impact by H-index

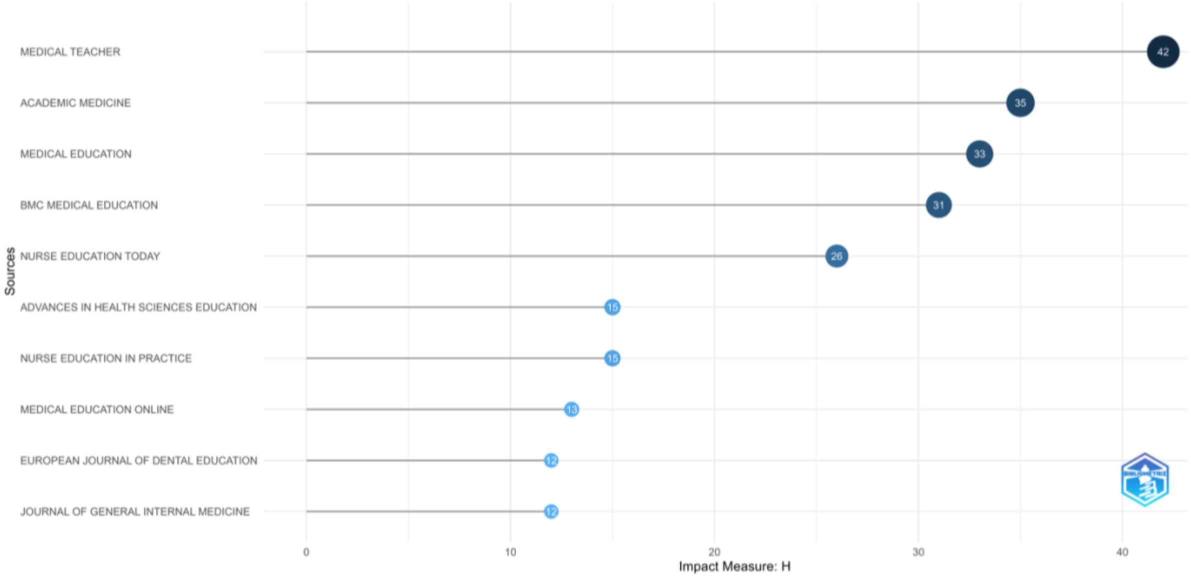


**Supplementary Material 13.** Sources local impact by TC index

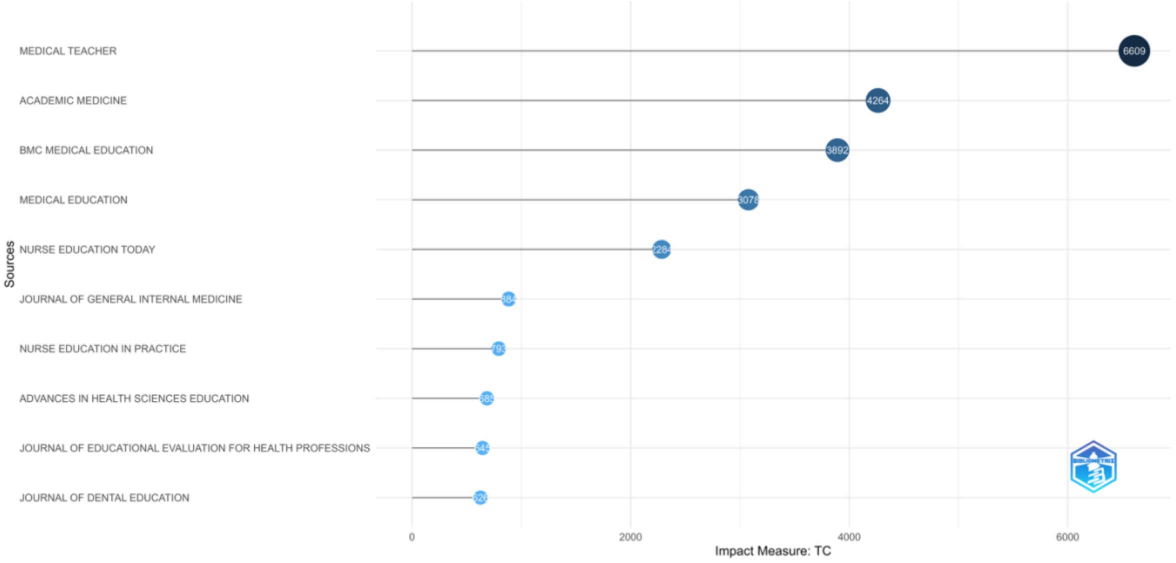


Abbreviations: TC, total citations.

**Supplementary Material 14.** Most globally cited documents

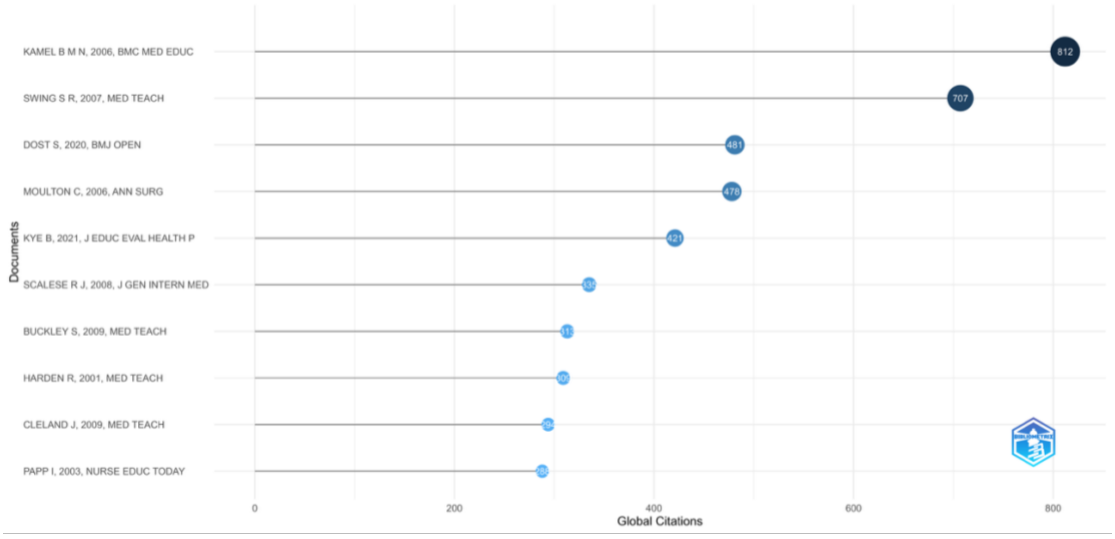


**Supplementary Material 15.** Most relevant authors

| No. | Authors | Affiliation | Articles | Articles Fractionalized |
|---|---|---|---|---|
| 1 | ROFF S | Centre for Medical Education, University of Dundee, Dundee, Scotland, UK. | 17 | 5.3004329 |
| 2 | SANTEN S A | University of Cincinnati: Cincinnati, Ohio, US | 14 | 2.1495782 |
| 3 | DORNAN T | School of Medicine, Dentistry and Biomedical Sciences, Queens University Belfast, Belfast, UK. | 13 | 3.56666667 |
| 4 | MCALEER S | University of Dundee, UK | 11 | 2.97619048 |
| 5 | BENNETT D | Medical Education Unit, University College Cork, National University of Ireland, Cork, Ireland | 9 | 2.14358974 |
| 6 | HAUER K E | Department of Medicine, University of California, San Francisco School of Medicine, San Francisco, California, USA | 9 | 1.89358974 |
| 7 | DYRBYE L | Department of Medicine, University of Colorado Anschutz Medical Campus, Aurora | 8 | 1.44775641 |
| 8 | FISCHER M | Institute for Medical Education, University Hospital, LMU Munich, Munich, Germany | 8 | 1.76785714 |
| 9 | TEHERANI A | University of California, School of Medicine, San Francisco, California | 8 | 1.5452381 |
| 10 | TOKUDA Y | Muribushi Okinawa Center for Teaching Hospitals, Urasoe Okinawa Japan. | 8 | 0.93015873 |
| 11 | VAN D V C | School of Health Professions Education, Faculty of Health, Medicine and Life Sciences, Maastricht University, Maastricht, the Netherlands | 8 | 1.27668067 |
| 12 | WATLING C | Royal College of Physicians and Surgeons of Canada, Ottawa, Ontario, Canada | 8 | 1.83333333 |

**Supplementary Material 16.** Author's local impact

| No. | Author | h-index | g-index | m-index | TC | NP | PY start |
|---|---|---|---|---|---|---|---|
| 1 | ROFF S | 13 | 21 | 0.5 | 1431 | 21 | 2000 |
| 2 | MCALEER S | 10 | 12 | 0.38461538 | 1051 | 12 | 2000 |
| 3 | DORNAN T | 9 | 13 | 0.36 | 540 | 13 | 2001 |
| 4 | BENNETT D | 8 | 9 | 0.5 | 227 | 9 | 2010 |

| | | | | | | | |
|---|---|---|---|---|---|---|---|
| 5 | SANTEN S A | 8 | 13 | 0.57142857 | 188 | 14 | 2012 |
| 6 | HAUER K E | 7 | 9 | 0.4375 | 233 | 9 | 2010 |
| 7 | SCHERPBIER A | 7 | 13 | 0.26923077 | 388 | 13 | 2000 |
| 8 | APPELBAUM N P | 6 | 6 | 0.66666667 | 172 | 6 | 2017 |
| 9 | CHANG Y | 6 | 8 | 0.54545455 | 111 | 8 | 2015 |
| 10 | KELLY M | 6 | 9 | 0.3 | 277 | 9 | 2006 |
| 11 | LINGARD L | 6 | 9 | 0.26086957 | 477 | 9 | 2003 |
| 12 | O'FLYNN S | 6 | 6 | 0.375 | 182 | 6 | 2010 |
| 13 | RIQUELME A | 6 | 7 | 0.35294118 | 350 | 7 | 2009 |
| 14 | TEHERANI A | 6 | 9 | 0.4 | 150 | 9 | 2011 |
| 15 | VAN D V C | 6 | 13 | 0.23076923 | 567 | 13 | 2000 |
| 16 | VAN D V C P M | 6 | 7 | 0.3 | 302 | 7 | 2006 |
| 17 | WATLING C | 6 | 9 | 0.42857143 | 485 | 9 | 2012 |

Abbreviations: TC, total citations; NP, number of publications; PY, publication year.

**Supplementary Material 17.** Authors local impact by H-index

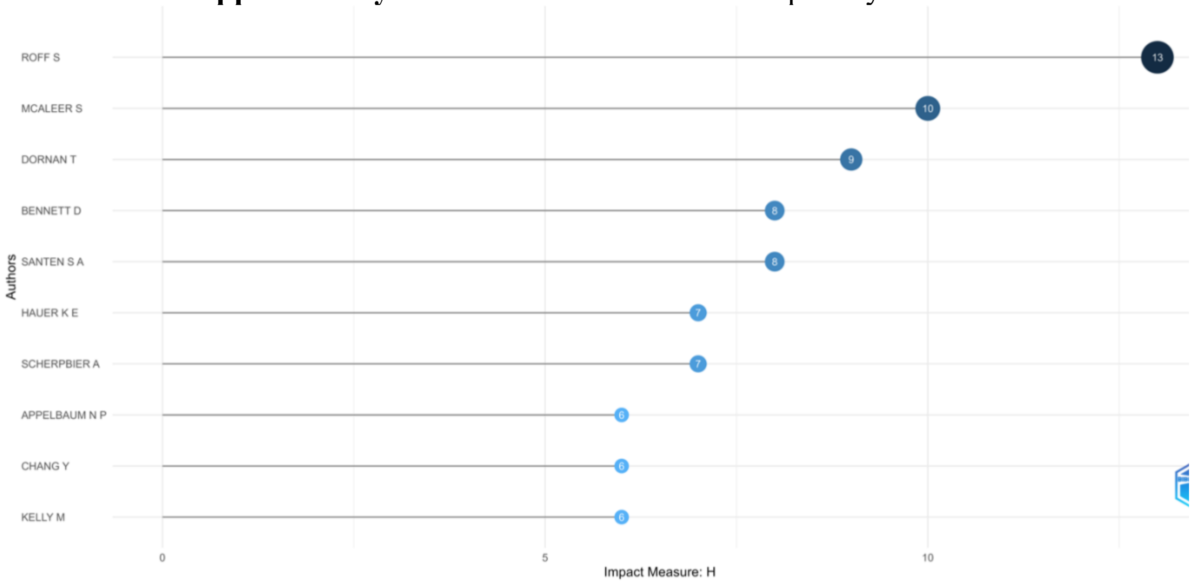


**Supplementary Material 18.** Author collaboration network

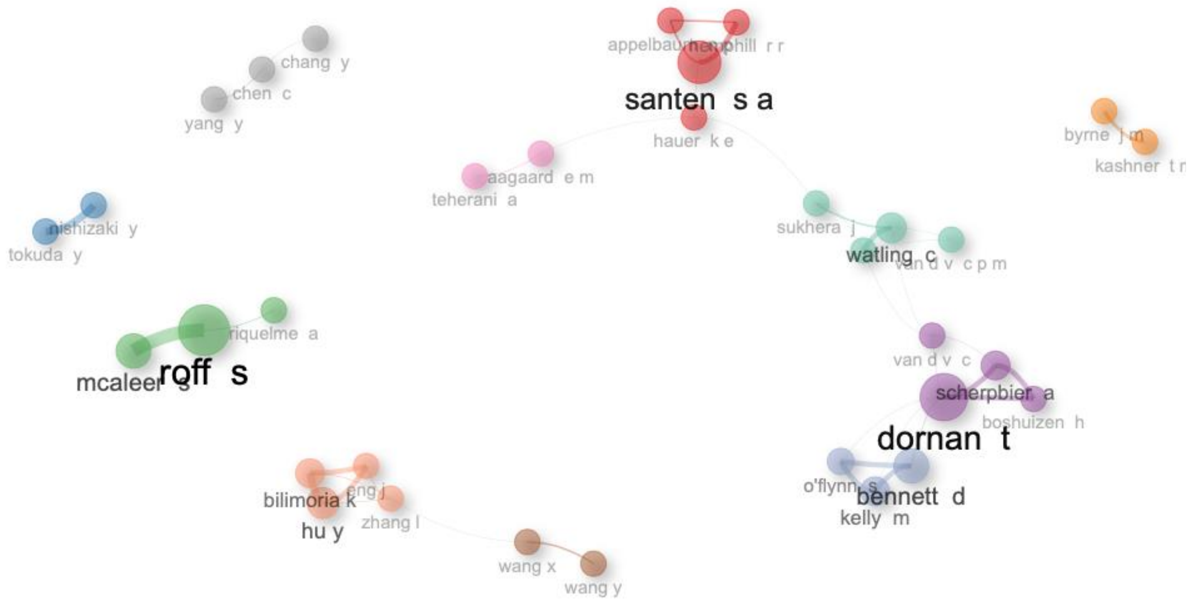


**Supplementary Material 19.** Author's production over time

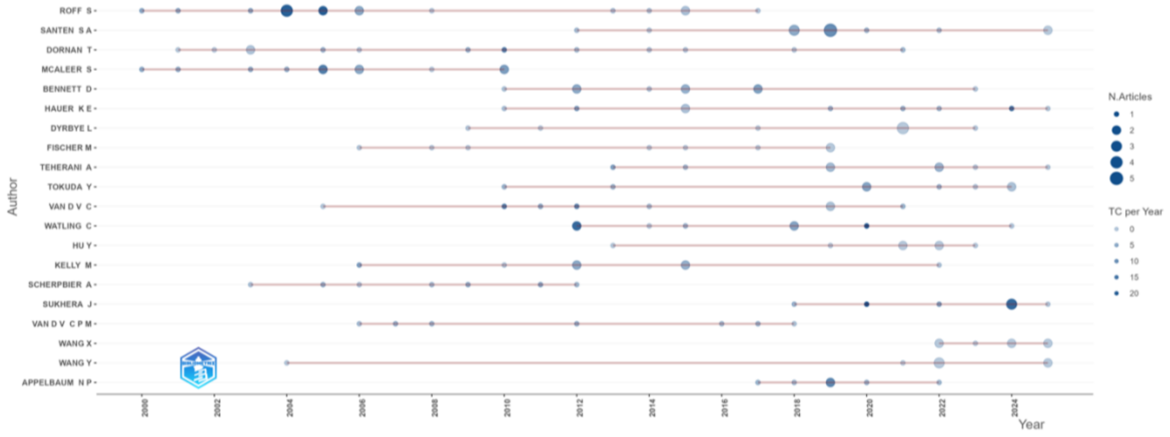


Abbreviations: N.Articles, number of articles; TC per Year, total citations per year.

**Supplementary Material 20.** Most relevant affiliations

| S.no. | Affiliates | Articles | Institutional breakdown |
|---|---|---|---|
| 1 | HARVARD UNIVERSITY | 322 | ▪ Harvard Medical School,<br>▪ Harvard School of Dental Medicine,<br>▪ Harvard Pilgrim Health Care,<br>▪ Harvard T.H. Chan School of Public Health,<br>▪ Harvard University Medical Affiliates. |
| 2 | UNIVERSITY OF CALIFORNIA | 283 | ▪ University of California (San Franciso, San Diego, Los Angeles, Irvine, Davis, Riverside), |

| | | | |
|---|---|---|---|
| | | | ▪ University of California System. |
| 3 | UNIVERSITY OF MICHIGAN | 176 | ▪ University of Michigan Medical School,<br>▪ University of Michigan System |
| 4 | UNIVERSITY OF WASHINGTON | 144 | ▪ University of Washington School of Medicine,<br>▪ University of Washington School of Dentistry,<br>▪ University of Washington School of Public Health. |
| 5 | UNIVERSITY OF TEXAS | 135 | ▪ University of Texas Health Science Center,<br>▪ University of Texas System,<br>▪ Dell Medical School at the University of Texas,<br>▪ University of Texas School Public Health. |
| 6 | UNIVERSITY OF TORONTO | 124 | ▪ University of Toronto,<br>▪ University of Toronto Temerty of Medicine. |
| 7 | UNIVERSITY OF PENNSYLVANIA | 116 | ▪ University of Pennsylvania,<br>▪ Hospital of the University of Pennsylvania,<br>▪ Perelman School of Medicine at the University of Pennsylvania. |
| 8 | BAYLOR COLLEGE OF MEDICINE | 95 | ▪ Baylor College of Medicine. |
| 9 | UNIVERSITY OF COLORADO | 92 | ▪ University of Colorado School of Medicine,<br>▪ University of Colorado System,<br>▪ University of Colorado School of Public Health. |
| 10 | JOHNS HOPKINS | 81 | ▪ Johns Hopkins University,<br>▪ Johns Hopkins Medicine,<br>▪ Johns Hopkins University School of Medicine,<br>▪ Johns Hopkins Hospital and Health System. |
| 11 | MAASTRICHT UNIVERSITY | 79 | ▪ Maastricht University. |
| 12 | UNIVERSITY OF OTAGO | 75 | ▪ University of Otago,<br>▪ The University of Otago College of Education. |
| 13 | MONASH UNIVERSITY | 74 | ▪ Monash University. |
| 14 | NORTHWESTERN UNIVERSITY | 74 | ▪ Northwestern University,<br>▪ Northwestern University Feinberg School of Medicine. |
| 15 | YALE | 74 | ▪ Yale School of Medicine,<br>▪ Yale University,<br>▪ Yale School of Public Health. |

**Supplementary Material 21.** Most frequent words

| Words | Occurrences |
|---|---|
| human | 3331 |
| female | 1336 |
| male | 1258 |
| learning | 1016 |
| adult | 953 |
| curriculum | 895 |
| medical education | 876 |
| article | 754 |
| clinical competence | 678 |
| education | 643 |
| medical student | 515 |
| surveys and questionnaires | 478 |
| questionnaire | 454 |
| teaching | 426 |
| perception | 425 |
| students medical | 418 |
| young adult | 386 |
| internship and residency | 353 |
| united states | 284 |
| qualitative research | 276 |
| learning environment | 263 |
| psychology | 259 |
| attitude of health personnel | 241 |
| cross-sectional studies | 240 |
| education medical undergraduate | 238 |
| medical school | 233 |
| students | 231 |
| educational measurement | 230 |
| procedures | 228 |
| cross-sectional study | 210 |
| controlled study | 205 |
| students nursing | 202 |
| human experiment | 188 |
| education medical graduate | 179 |
| nursing student | 179 |
| education medical | 175 |
| program evaluation | 171 |
